# MKK3 sustains cell proliferation and survival through p38DELTA MAPK activation in colorectal cancer

**DOI:** 10.1038/s41419-019-2083-2

**Published:** 2019-11-06

**Authors:** Lorenzo Stramucci, Angelina Pranteda, Arianna Stravato, Carla Azzurra Amoreo, Annarita Pennetti, Maria Grazia Diodoro, Armando Bartolazzi, Michele Milella, Gianluca Bossi

**Affiliations:** 1 Laboratory of Medical Physics and Expert Systems, Department of Diagnostic Research and Technological Innovation, IRCCS - Regina Elena National Cancer Institute, 00144 Rome, Italy; 2 Oncogenomic and Epigenetic Unit, Department of Diagnostic Research and Technological Innovation, IRCCS - Regina Elena National Cancer Institute, 00144 Rome, Italy; 3 Department of Pathology, IRCCS - Regina Elena National Cancer Institute, 00144 Rome, Italy; 4 Pathology Research Laboratory, Sant’Andrea Hospital, 00189 Rome, Italy; 5 Medical Oncology 1, IRCCS - Regina Elena National Cancer Institute, 00144 Rome, Italy; 6 Present Address: Oncology Section, Department of Medicine, University of Verona School of Medicine/Verona University Hospital Trust, 37134 Verona, Italy

**Keywords:** Kinases, Colon cancer, Mechanisms of disease, Target identification, Phosphorylation

## Abstract

Colorectal cancer (CRC) is one of the most common malignant tumors worldwide and understanding its underlying molecular mechanisms is crucial for the development of therapeutic strategies. The mitogen-activated protein kinase-kinase 3 (MKK3) is a specific activator of p38 MAP kinases (p38 MAPKs), which contributes to the regulation of several cellular functions, such as proliferation, differentiation, apoptosis as well as response to drugs. At present, the exact MKK3/p38 MAPK pathway contribution in cancer is heavily debated because of its pleiotropic function. In this work, we retrospectively explored the prognostic and pathobiologic relevance of MKK3 in a cohort of CRC patients and assessed MKK3 molecular functions in a panel of CRC lines and colonocytes primary cultures. We found increased MKK3 levels in late-stage CRC patients which correlated with shorter overall survival. Herein, we report that the MKK3 targeting by inducible RNA interference univocally exerts antitumor effects in CRC lines but not in primary colonocytes. While MKK3 depletion per se affects growth and survival by induction of sustained autophagy and death in some CRC lines, it potentiates response to chemotherapeutic drug 5-fluorouracil (5-FU) in all of the tested CRC lines in vitro. Here, we demonstrate for the first time that in CRC the MKK3 specifically activates p38delta MAPK isoform to sustain prosurvival signaling and that such effect is exacerbated upon 5-FU challenge. Indeed, p38delta MAPK silencing recapitulates MKK3 depletion effects in CRC cells in vitro and in vivo. Overall, our data identified a molecular mechanism through which MKK3 supports proliferation and survival signaling in CRC, further supporting MKK3 as a novel and extremely attractive therapeutic target for the development of promising strategies for the management of CRC patients.

## Introduction

Colorectal cancer (CRC) is the third most common cancer, the fourth most common cause of cancer death, and the second most common cancer in terms of number of individuals living with cancer 5 years after diagnosis worldwide^1^. CRC is a complex disease with a variable clinical course, even in tumors with similar histopathological features, with 33% of recurrence in stages II and III and 73% in metastatic stage IV CRC patients undergoing potentially curative resections^2^. Therefore, the identification of new molecular targets for the development of novel, more efficient therapeutic strategies is imperative.

The mitogen-activated protein kinase-kinase 3 (MKK3) belongs to a dual specificity kinase group (MKK) and is activated by a wide array of upstream kinases (MEKK1–4) through Ser-189 and Thr-193 phosphorylation^3^. MKK3 serves, together with MKK6, as a specific activator of p38 mitogen activated protein kinase (MAPK)^4–6^.

The p38 MAPK pathway is activated upon a variety of stimuli, including cytokines and growth factors, cellular stress, and irradiation, to respond accordingly, resulting into different effects depending on the type, duration, and intensity of the triggering cues^7–11^. Four separate p38 MAPK isoforms (alpha, beta, gamma, and delta), encoded by four different genes, have been described^8,12^. The different isotypes display overlapping functions, although a consistent degree of preferential interaction with upstream mediators and downstream targets appears to be fundamental for fine-tuning of key processes^5,6,13,14^. As a consequence, despite a quite extensive detailing of precise signaling events, the exact contribution of p38 MAPK activation in cancer is still heavily debated and a controversial role has been reported for many members of the p38 MAPK-signaling pathway^8,9,11,15,16^.

We have previously shown that MKK3 could represent an attractive therapeutic target in different types of carcinoma^4,17,18^: however, because of the pleiotropic nature of p38 MAPK pathway, the final biological outcome of MKK3 targeting could greatly vary according to cell-context.

In this work, we found that MKK3 is highly expressed in advanced stages of CRC patients and correlates to poor prognosis. Taking advantage of a panel of human CRC lines and primary colonocytes, we dissected the MKK3 molecular and functional role in CRC and unveiled MKK3 as an oncogenic mediator and excellent candidate for perspective targeting in CRC. Strikingly, while MKK3 targeting resulted into sustained autophagy induction and impaired cell growth, as we previously reported^17^, only in a subset of CRC lines, it strictly potentiated 5-fluorouracil (5-FU) anti-tumor effects in all of the tested CRC lines. Mechanistically, MKK3 depletion-mediated 5-FU boosting depended on the blockade of drug-induced MKK3/p38delta MAPK-transduced prosurvival signaling in CRC cells. Interestingly, MKK3 blockade in primary colonocytes did not result in toxic effects, indicating MKK3 targeting could represent a selective, safe, and effective tool in the management of CRC.

## Results

### MKK3 is highly expressed in late stages CRC and correlates with poor overall survival

**Fig. 1:**
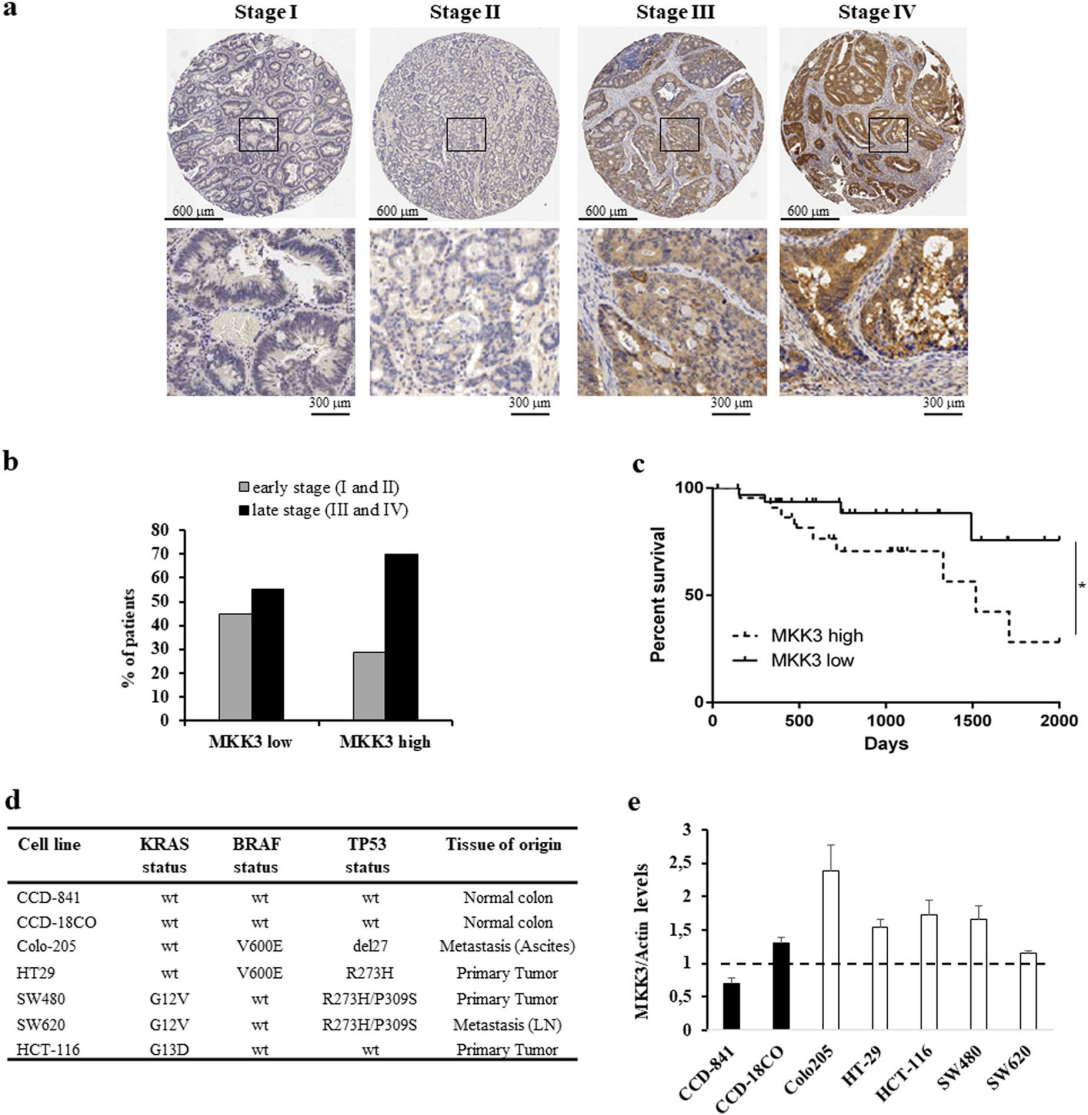
MKK3 is highly expressed in CRC patients with advanced stages of disease, and correlates to poor patient prognosis. **a** Representative cases of histologic details of MKK3 positivity on retrospective TMA CRC patient cohort. **b** Histogram of recorded MKK3 positivity in early (stages I and II) (*n* = 68) and late stage (stages III and IV) (*n* = 110) retrospective TMA CRC patient cohort (see Supplementary Table 1); Fisher’s exact test *p = 0.043. **c** Kaplan–Meier survival analysis of high MKK3 expressing (*n* = 24) and low MKK3 expressing (*n* = 33) colon adenocarcinoma (COAD) patients in the TCGA database; Log-rank (Mantel–Cox) test *p = 0.0487. **d** Panel of adopted CRC line and primary colonocytes with description of the genetic background of clinically relevant KRAS, BRAF, and TP53 status and tissue of origin reported for each cell line. **e** MKK3 levels in the adopted CRC lines and colonocyte primary cultures panel. Protein levels were determined by western blot and normalized to relative loading control actin for each CRC and primary culture. Dashed line represent average expression levels of MKK3 in primary colonocytes. Results are reported as mean and S.D. of two independent analyses performed; unpaired *t*-test. *p = 0.012

MKK3 has been previously reported to be either upregulated or downregulated in different types of cancers^4,19,20^. In order to evaluate perspective appropriateness of MKK3 targeting in CRC patients, we performed IHC analysis on a tissue micro-array (TMA) retrospective cohort of n.178 CRC patients (Supplementary Fig. 1). Different degrees of MKK3 positivity were found in the tumor samples (Fig. 1a), reflecting different MKK3 levels of expression. Stratification for early (stage I or II) versus late (stage III or IV) tumor stages revealed that MKK3 is preferentially and significantly highly expressed in late stage disease (*p* = 0.043) (Fig. 1b and Supplementary Table 1). Also, analysis of publicly available Cancer Genome Atlas (TCGA) Datasets, revealed that a higher expression of MKK3 is correlated to significantly poorer prognosis as compared to low MKK3 expression in CRC patients (*p* = 0.0487) (Fig. 1c and Supplementary Table 2), supporting the prognostic and pathobiologic relevance for MKK3 in CRC.

### MKK3 targeting induces autophagy in CRC lines but not in primary colonocytes

**Fig. 2:**
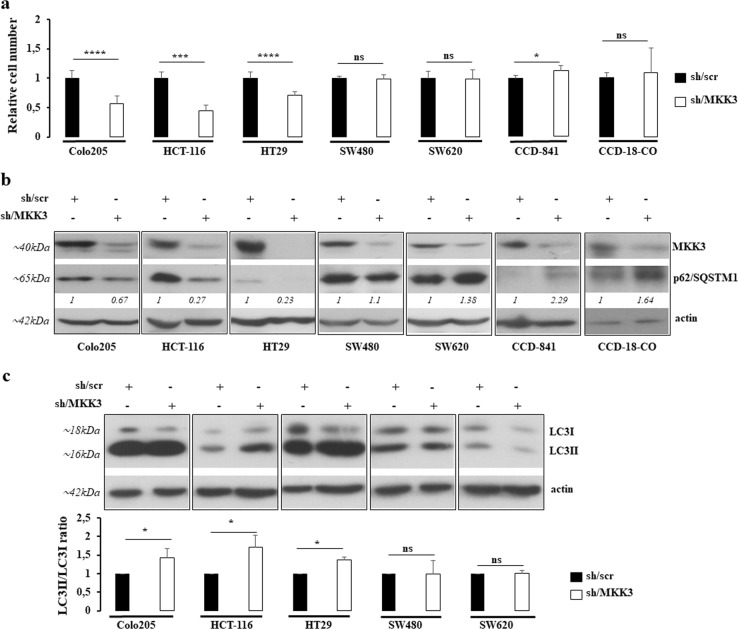
MKK3 depletion affects cell growth via induction of autophagy in a subset of CRC lines. **a** Relative cell number of sh/scr and sh/MKK3 sublines after 144 h doxycycline (1 μg/ml) treatment were determined by trypan blue exclusion assay and numbers of viable cells reported as mean and S.D. of three independent experiments. Significance was analyzed using unpaired *t*-test. Results from each sh/MKK3 subline were normalized to those of relative control (sh/scr subline) set to 1.0. **b** Western blot analyses were performed on protein lysates derived from sh/scr and sh/MKK3 sublines after 144 h doxycycline (1 μg/ml) treatment. Blots were incubated with indicated antibodies. More relevant bands from the same filter at same exposure length are reported. Representative results of three independent experiments are reported. **c** LC3II/LC3I ratio and data are reported as mean and S.D. of three independent experiments. Densitometry was performed with ImageJ software and quantified with respect to controls (sh-scr) set to 1.0. Significance was analyzed using unpaired *t*-test. *p < 0.05, ***p < 0.001, ****p < 0.0001, n.s.: not significant

To functionally validate MKK3 as a therapeutic target, we selected a panel of CRC lines differing for BRAF, KRAS, and TP53 status and primary colonocytes (Fig. 1d). The evaluation of MKK3 protein levels by Western Blot revealed a tendency towards increased MKK3 levels in CRC lines when compared to primary colonocytes (Fig. 1e and Supplementary Fig. 1), suggesting that dysregulation of MKK3 signaling could be tumor-tissue selective. In order to assess the functional requirement of MKK3 in CRC, selected cancer lines were engineered with a doxycycline-inducible RNA interference (RNAi) system^17^ generating sh/scr and sh/MKK3 sublines. Efficient MKK3 depletion per se (Fig. 2b) resulted into a decrease in cell growth and increased cell death in HT29, HCT-116, as already reported^17,18^, and Colo205 (Fig. 2a, and Supplementary Fig. 2). Contrarily, no significant effects on cell growth and cell survival were observed in SW480 and SW620 lines upon MKK3 depletion (Fig. 2a and Supplementary Fig. 2). At the molecular level, in agreement with our previous reports^17,18^, the MKK3 silencing functional effects correlated with a decrease in the late autophagosome marker p62/SQSTM1 levels (Fig. 2b) and an increased LC3II/LC3I ratio (Fig. 2c), indicating that, in line with our previous report^17^, triggering of sustained autophagy is responsible for the growth impairment in MKK3 depletion-sensitive CRC sublines. Accordingly, no altered p62/SQSTM1 levels and LC3II/LC3I ratio were observed in sh/MKK3 SW480 and SW620 sublines when compared to relative controls (sh/scr) (Fig. 2b, c).

Furthermore, we previously reported that MKK3 silencing did not affect normal fibroblast (FB1329) and epithelial (MCF-10A) primary cultures^17^. In order to better assess lack of toxicity on normal counterpart, we molecularly and functionally explored the effects induced upon MKK3 silencing in the two different colonocytes primary cultures (CCD-841 and CCD-18CO). Indeed, in this setting, MKK3 silencing (Fig. 2b) per se was not only well tolerated by both tested primary cultures, but also appeared to slightly sustain their proliferation (Fig. 2a). Moreover, in sharp contrast with CRC lines, we detected accumulation of p62/SQSTM1 levels in colonocytes (Fig. 2b). Overall results are confirmatory that cancer cells, but not their normal counterpart, rely on the exacerbation of the MKK3-signaling cascade to sustain proliferation and survival.

### 5-FU activates MKK3-signaling pathway in CRC lines

**Fig. 3:**
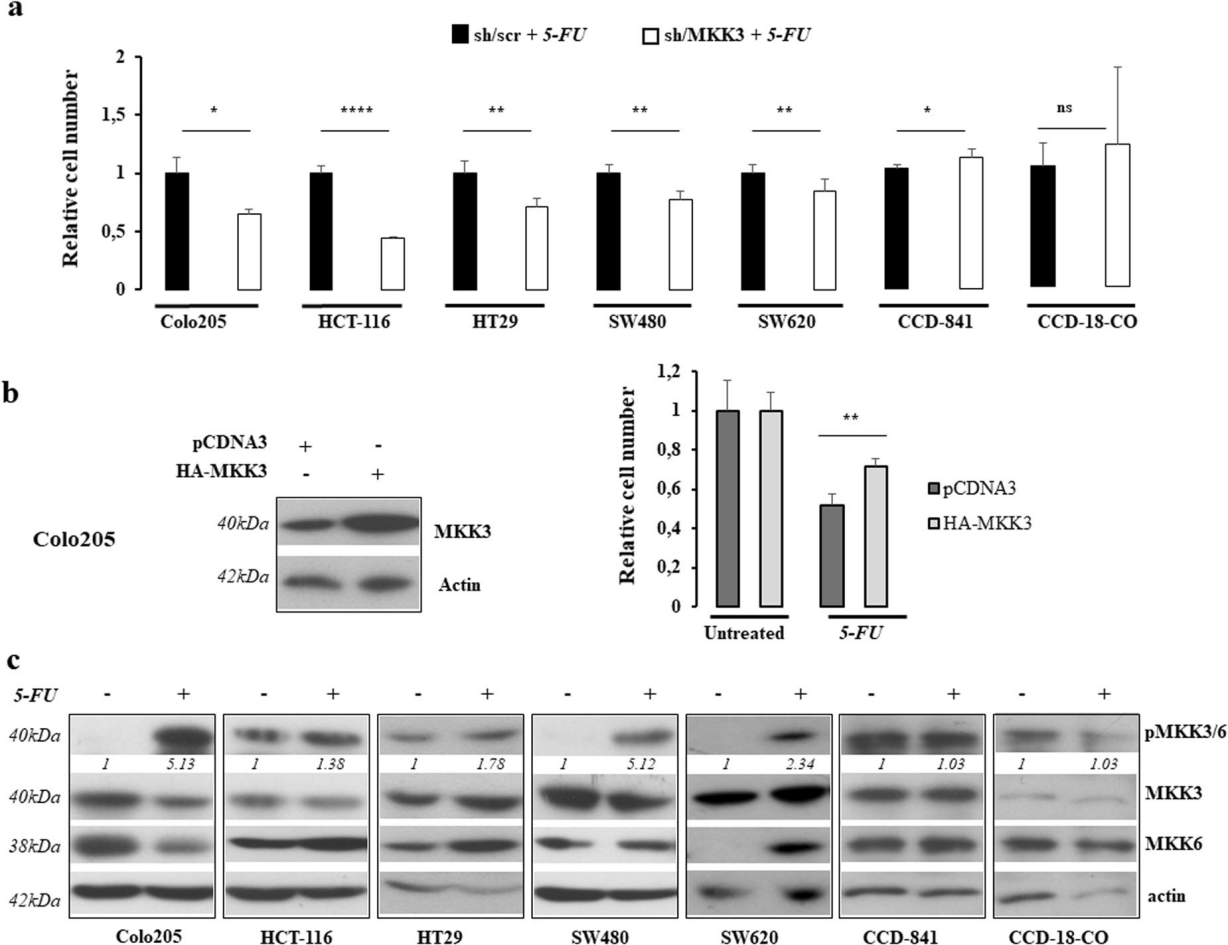
5-FU activates MKK3 in CRC but not in primary colonocytes. **a** Relative cell number of sh/scr and sh/MKK3 CRCs sublines after 5-FU IC50 treatments as described in “Materials and methods”. Results are reported as mean and S.D. of three independent experiments, and effects in sh/MKK3 + 5-FU condition normalized to relative controls (sh/scr + 5-FU) set to 1.0. Significance was analyzed using unpaired *t*-test. **b** Left panel: Western blot on protein lysates of pCDNA3 and pCDNA3-HA-MKK3 transiently transfected Colo205 cells. Blots were incubated with indicated antibodies. More relevant bands from the same filter at same exposure length are reported. Right panel: Relative live cell number in pCDNA3 and pCDNA3 HA-MKK3 cells untreated or 5-FU treated. Data are reported as mean and S.D. of two independent experiments, where HA-MKK3 + 5-FU condition were normalized to those of relative control pCDNA3 + 5-FU set to 1.0. Significance was analyzed using unpaired *t*-test. **c** Western Blot analyses were performed on protein lysates derived from cell lines exposed or not to 5-FU. Blots were incubated with indicated antibodies. Representative results of three independent experiments are reported; Densitometry was performed with ImageJ software and relative band intensity was normalized to β-actin and quantified with respect to untreated controls set to 1.0; more relevant bands from the same filter at same exposure length are reported. *p < 0.05, **p < 0.01, ****p < 0.0001, n.s.: not significant

We previously claimed that MKK3 depletion potentiates chemotherapeutics (5-FU, Adriamycin) effects in cancer lines of different histotypes allowing drug-dose reduction^17^. Accordingly, we investigated the response to 5-FU, the cornerstone of treatment for CRC patients^21^, in combination with MKK3 targeting in our panel of CRC lines. To this aim, we first determined for each CRC line the 5-FU dosage corresponding to the half-maximal inhibitory concentration (IC50) (Supplementary Fig. 3) to be adopted in co-treatment studies. Engineered CRC sh/scr and sh/MKK3 sublines were pretreated with doxycycline (DOX) to induce sh/RNA expression, and thereafter challenged with 5-FU IC50. The 5-FU exposure when combined with MKK3 silencing resulted into boosting of the chemotherapeutic effects in all of the tested CRC lines (Fig. 3a), including those insensitive to MKK3 depletion-induced autophagy (SW480, SW620) (Fig. 3a). In accordance with achieved results, the exogenous MKK3 expression attenuated 5-FU response in Colo205 cells (Fig. 3b). These results indicate that MKK3 hinders response to 5-FU and that mechanisms other than triggering of sustained autophagy might underlie such effect. In order to dissect how MKK3 affects the 5-FU response in CRC, we investigated the MKK3 signaling upon drug exposure. Indeed, consistently with previous reports^22^, 5-FU treatment increases levels of phosphorylated MKK3 in all of the tested CRC lines (Fig. 3c). Of interest, in primary colonocytes, the 5-FU exposure, at a dosage determined for each culture (Supplementary Fig. 4), did not induce the MKK3 pathway activation (Fig. 3c) and the MKK3 depletion in these cultures showed a tendency to protect from 5-FU antiproliferative effects (Fig. 3a).

### The MKK3 pro-survival signaling relies on p38delta MAPK activation

**Fig. 4:**
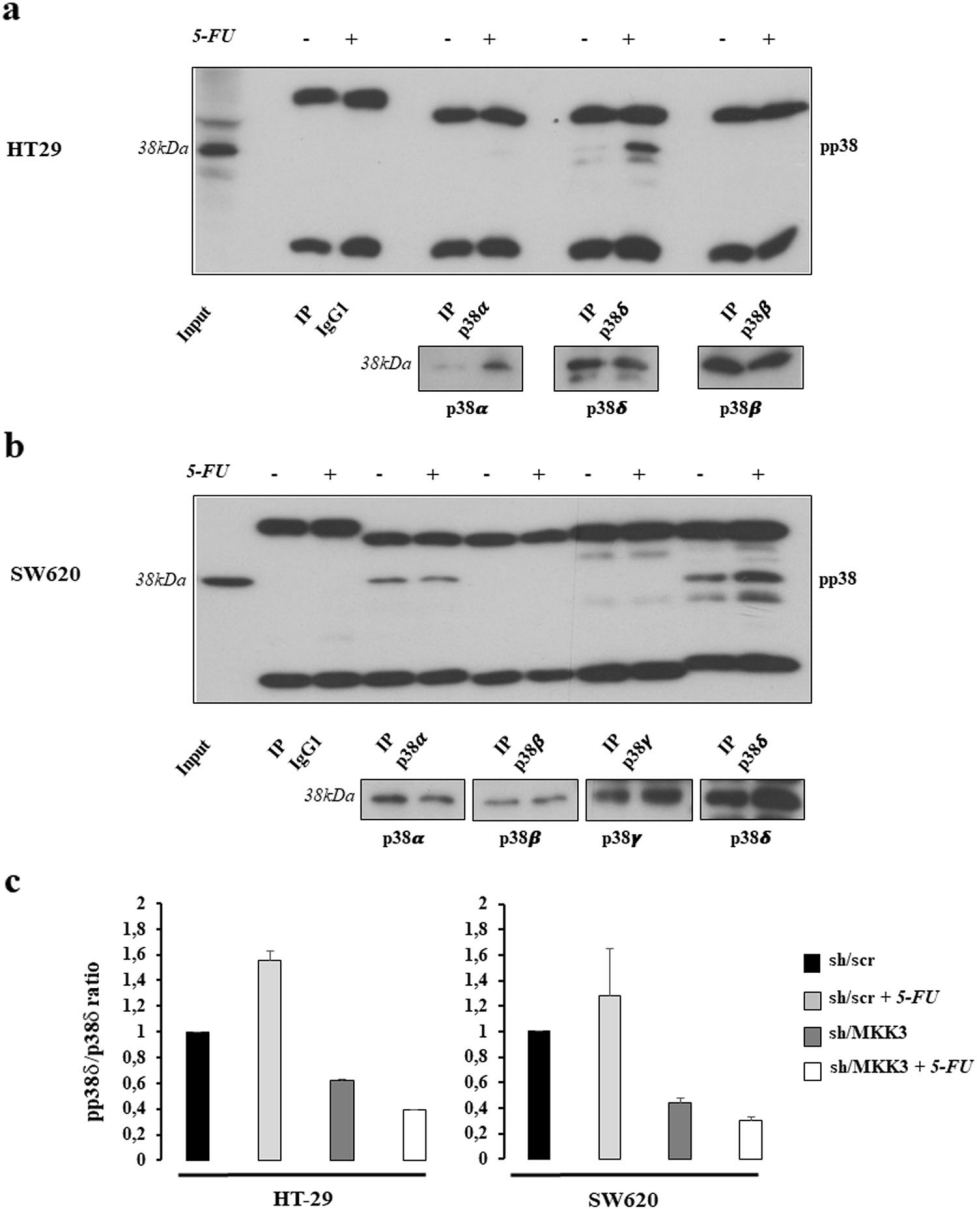
Activated MKK3 selectively phosphorilates p38delta MAPK. **a**, **b** Western Blot analysis for phospho p38 levels in p38 isoform-specific immunoprecipitates from HT29 (**a**) and SW620 (**b**) lines. Total levels of precipitated isoforms are shown as insets; more relevant bands from the same filter at same exposure length are reported. **c** Densitometric analysis of phospho p38 MAPK levels in p38delta-immuneprecipitates of sh/scr and sh/MKK3 HT29 (left) and SW620 (right) cells exposed or not to 5-FU. Data are reported as mean and SD of results from two independent experiments. Densitometry was performed with ImageJ software and the relative pp38/p38 MAPK ratio was normalized to sh/scr untreated control set to 1.0

In order to assess the underlying molecular mechanisms involved in 5-FU response in CRC, we further investigated whether 5-FU through MKK3 might affect activation of specific p38 MAPK isoforms in CRC. Accordingly, each isotype was immune-precipitated from total cell lysates derived from 5-FU treated and untreated HT29 or SW620 cells and the phosphorylation status assessed by immunoblotting. Among the p38 MAPK proteins, the isoform delta was highly phosphorylated upon 5-FU exposure in both tested CRC lines (Fig. 4a, b). In addition, the 5-FU-induced p38delta MAPK phosphorylation was strictly dependent on MKK3, since its depletion reduced the phosphorylation levels of the p38delta MAPK isoform, with respect to controls (sh/scr), in untreated conditions and upon 5-FU treatments (Fig. 4c and Supplementary Fig. 5).

**Fig. 5:**
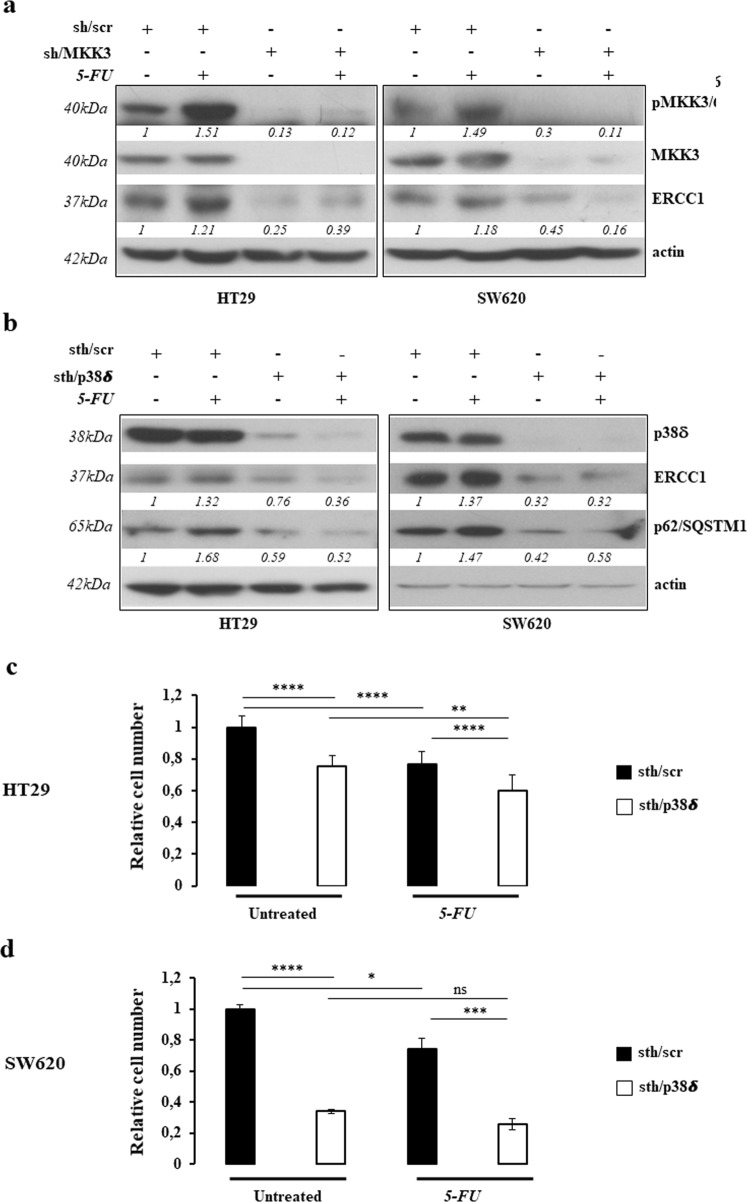
MKK3 affects 5-FU efficacy via activation of p38delta MAPK. **a**, **b** Western Blot on protein lysates from sh/scr and sh/MKK3 HT29 and SW620 sublines (**a**) and sth/scr and sth/p38δ transfected HT29 and SW620 cells (**b**) either untreated or exposed to 5-FU. Blots were incubated with indicated antibodies. Representative results of three independent experiments are reported; more relevant bands from the same filter at same exposure length are reported. Densitometry was performed with ImageJ software and relative band intensity was normalized to β-actin and quantified with respect to sh/scr controls set to 1.0. **c**, **d** Relative cell number of sth/scr and sth/p38δ transduced HT29 (**c**) and SW620 (**d**) cells untreated and upon 5-FU exposure. Data are reported as mean and S.D. of results from three independent experiments and analyzed using unpaired *t*-test. **p < 0.01, ***p < 0.001, ****p < 0.0001

Molecularly, the p38 MAPK activation has been previously reported to induce pro-survival effects, upon etoposide treatment in NSCLC^23^ and 5-FU in gastric cancer^24^, via upregulation of excision repair cross-complementary 1 (ERCC1) protein, one of the critical proteins involved in nuclear excision repair (NER). Hence, we investigated whether in CRC lines the ERCC1 upregulation might constitute one of the pro-survival cues downstream of MKK3/p38delta MAPK signaling cascade. Consistently, abrogation of ERCC1 expression was revealed in MKK3 depleted HT29 and SW620 cells in untreated conditions and upon 5-FU treatment (Fig. 5a). To investigate the functional role of activated p38delta MAPK isoform in MKK3-mediated pro-survival signaling, we assessed the response to p38delta MAPK depletion in HT29 and SW620 cells. Stealth RNAi efficiently depleted p38delta MAPK in both tested CRC lines without affecting the other p38 MAPK isoforms expression levels (Supplementary Fig. 6). The p38delta MAPK knockdown mimicked the MKK3 depletion effects by: (i) inducing authophagic response, as assessed by decreased p62/SQSTM1 levels (Fig. 5b); (ii) reducing the ERCC1 protein levels (Fig. 5b); (iii) impairing cell growth not only in HT29 (Fig. 5c), but also in SW620 (Fig. 5d) cells, which displayed a resistant phenotype to MKK3 silencing; (iv) boosting significantly response to 5-FU in HT29 cells (Fig. 5c). Furthermore, specific ERCC1 depletion, by small interference RNA (si/RNA), significantly affects cell proliferation and survival (Supplementary Fig. 7), further assessing that MKK3-signaling pathway sustains pro-survival effects through the ERCC1-positive regulation. Overall results prompt to support that the MKK3/p38delta MAPK-signaling pathway likely sustains pro-survival effects through ERCC1-positive regulation in CRC cells and suggest that, in line with recent report^25^, the p38delta MAPK isoform represent a key molecular player also in the control of the autophagic process.

### The p38delta MAPK sustains tumor malignancy in vivo

**Fig. 6:**
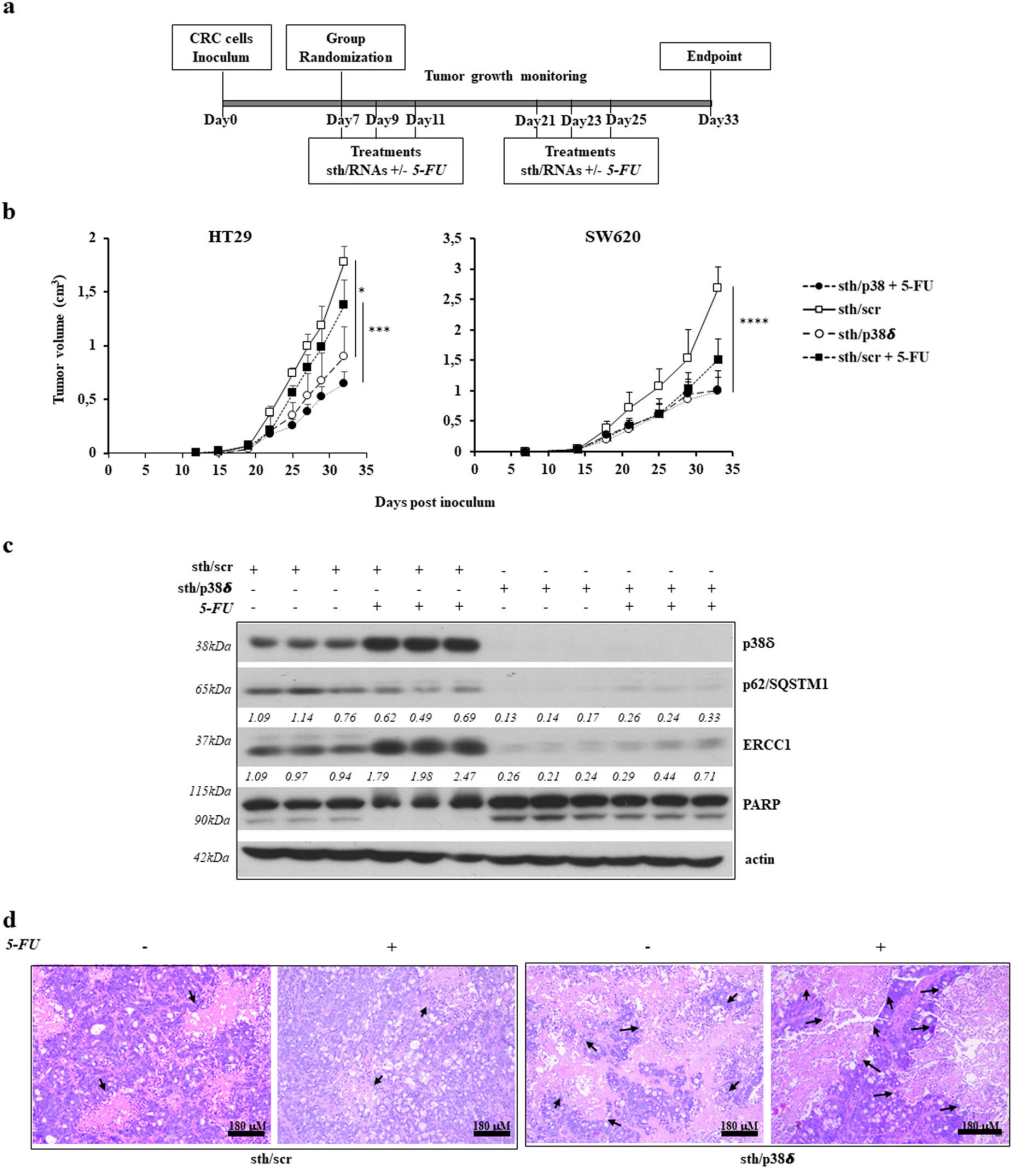
p38delta MAPK depletion recapitulates sh/MKK3 effects in vivo. **a** Representative diagram of the in vivo experiment. **b** In vivo growth (mean and SD) of HT29 and SW620 tumors according to the different treatments. **c** Western Blot analysis for p38delta MAPK, p62/SQSTM1, ERCC1, and PARP levels in sth/scr or sth/p38delta (alone and in combination with 5-FU) treated HT29 tumors. Actin was used as a normalizer; more relevant bands from the same filter at same exposure length are reported. Densitometry was performed with ImageJ software and relative band intensity was normalized to β-actin and quantified with respect to the average of sh/scr-untreated controls set to 1.0. **d** Representative H&E images of HT29 tumors in mice treated with sth/scr or sth/p38delta oligonucleotides alone or in combination with 5-FU. Black arrows indicate necrotic areas. Data were analyzed using two-way ANOVA. *p < 0.05, ***p < 0.001, ****p < 0.0001, n.s.: not significant

We have previously shown that MKK3 targeting counteracts HT29 tumor growth and boosts 5-FU efficacy in vivo^17^. Also, in agreement to in vitro data, MKK3 depletion boosts 5-FU efficacy in SW620 xenograft tumors (Supplementary Fig. 8). In order to verify the involvement of p38delta MAPK isoform in established sh/MKK3-mediated antitumor effects in vivo, we tested whether its specific targeting in HT29 and SW620-derived xenograft tumors might counteract malignancy and boost 5-FU effects. To deplete p38delta MAPK isoform in vivo, specific stealth RNAi (sth/p38δ) or controls (sth/scr) were subcutaneously injected into peritumoral region of xenograft lesions following defined schedule of treatment (Fig. 6a). Effect on tumor growth were followed weekly. Results revealed that with respect to control tumors (sth/scr), the p38delta MAPK depletion impairs tumor growth in both xenografted CRC models (HT29, SW620) (Fig. 6b). Furthermore, although p38delta MAPK targeting in combination with 5-FU resulted in stronger antitumor effects as compared to 5-FU alone (Fig. 6b), we found that p38delta MAPK targeting alone exerted similar antitumor effects regardless of the combination with 5-FU. At molecular level, efficient p38delta MAPK depletion was achieved in the sth/p38delta-treated tumors (Fig. 6c). In agreement with the in vitro data, the p38delta MAPK silencing consistently induced autophagy, as assessed by significant reduction of p62/SQSTM1, impaired pro-survival pattern, as assessed by reduced ERCC1 protein levels, and induced cell death, as assessed by increased PARP cleavage in the tumor tissue (Fig. 6c). Of interest, 5-FU treatments induce in vivo higher effects on ERCC1 protein levels when compared to those observed in vitro (Fig. 5b), which effects were massively abolished along with p38delta MAPK isoform depletion (Fig. 6c). Consistently, histologic analysis of tumor tissue, revealed vast areas of tumor necrosis in p38delta MAPK-depleted tumors (sth/p38δ 50%; sth/p38δ + 5FU 57.5%) in spite of a smaller tumor volume, as compared to control sth/scr tumors (sth/scr 28.7%; sth/scr + 5FU 32.5%) (Fig. 6d and Supplementary Table 3).

Altogether, these results confirm that p38delta MAPK isoform contributes to MKK3-transduced prosurvival signaling in vivo.

## Discussion

The role of p38 MAPK signaling in cancer is highly controversial with reports of both tumor promoting and impairing activities in different types of cancers^13–16,22,26–31^: while this is relatively unsurprising because of the intrinsic pleiotropic nature of p38 MAPK^8,15^, it also poses serious challenges at translating the manipulation of the p38 MAPK pathway for clinical purposes. Accordingly, in this work we dissected the contribution of the p38 MAPK-activating kinase MKK3 into several different CRC lines, unveiling a clear tumor suppressive activity of MKK3 targeting. Strikingly, in accordance with p38 MAPK involvement in many different cellular processes, it appears that MKK3 contribution to CRC tumor growth is manifold: in fact, we observed a cancer cell-line-specific dependence on MKK3, with autophagy being induced only in a subset of CRC lines upon MKK3 depletion, while this caused potentiation of 5-FU-induced killing in all of the tested CRC lines indicating that MKK3 inhibition exerts tumor-suppressive effects also by affecting mechanisms other than autophagy induction.

**Fig. 7:**
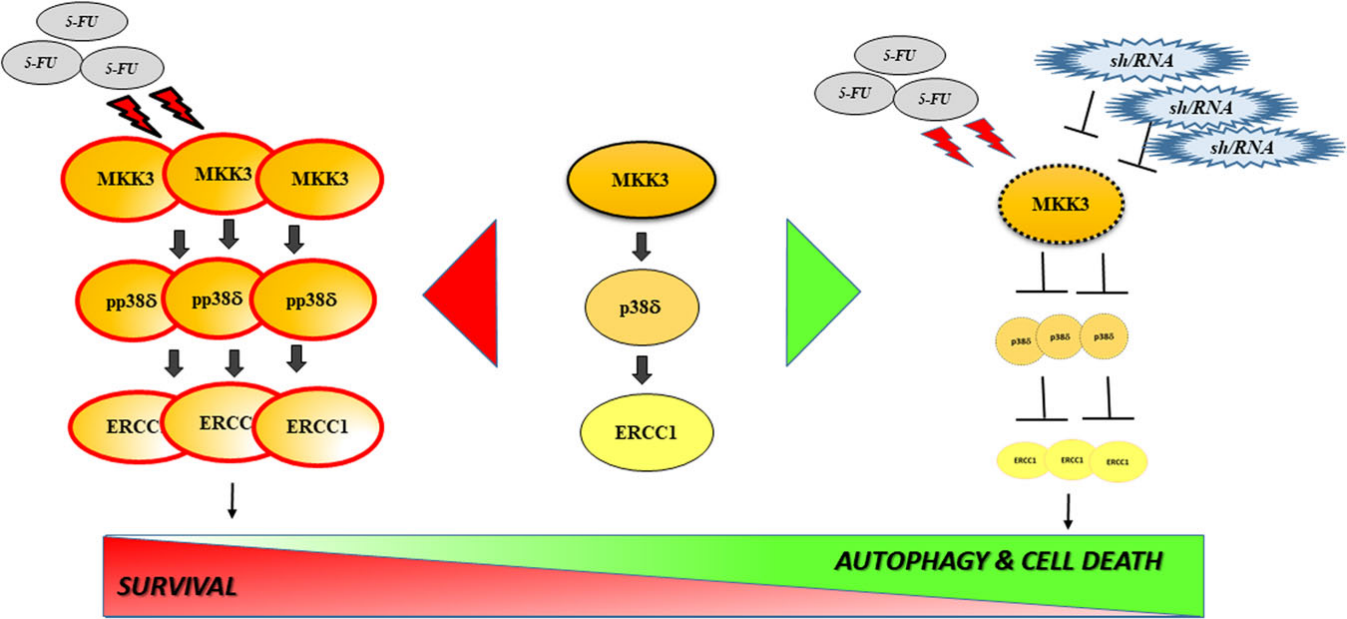
MKK3 as an actionable target sustaining 5-FU-triggered pro-survival signaling in CRC. Sketch illustrating how the MKK3/p38delta/ERCC1 pro-survival signaling is exacerbated in CRC in response to 5-FU exposure (left side). Targeting MKK3 prevents activation of 5-FU-triggered pro-survival signaling, priming CRC cells for sustained autophagy and drug-induced killing (right)

Mechanistically we found that 5-FU efficacy boosting upon MKK3 depletion depends, at least in part, on the blockade of 5-FU exposure-triggered MKK3/p38 MAPK-mediated activation of downstream pro-survival mediator ERCC1, involved in DNA repair (as summarized in Fig. 7). While p38 MAPK-mediated activation of ERCC1 has been previously reported^23^ and associated with pro-survival effects of activation of this pathway^32^, also the opposite effect of p38 MAPK activation has been reported and specifically linked to 5-FU-mediated induction of apoptosis via activation of p38alpha MAPK in CRC lines^22^. In this study, we demonstrated that in CRC p38delta MAPK is induced, and indeed highly phosphorylated by MKK3 as a result of 5-FU exposure, indicating that, beyond p38 MAPK alpha-mediated induction of apoptosis, also MKK3/p38delta MAPK pro-survival signaling is further triggered upon drug treatment. This simultaneous triggering of pro-apopototic and anti-apopototic mediators through the same cascade goes well along with the pleiotropic and controversial role reported for p38 MAPK, and underlines the need of a deeper characterization and validation of the exact contribution of each MAPK pathway member in physiologically representative models in which fine-tuning of this pathway is not hampered by target overexpression. In this perspective, our finding that p38delta MAPK targeting is able to impair p38 MAPK-mediated pro-survival effects, together with the previous report of p38alpha MAPK mediating instead proapoptotic effects^22^, points toward selective inhibition of specific isoforms: indeed, p38 MAPK inhibitors reached clinical testing and demonstrate a relative degree of isoform-specificity^33–35^. However, a deeper focus to target-specific isoforms while leaving others unaffected could actually offer the possibility of increasing drug efficacy and minimizing side-effects^36^. Nevertheless, developing isoform selective drugs for tightly related proteins, each having pleiotropic effects, is a long and complicated path not only in terms of selectivity but also as it concerns drug-safety. Interestingly, in such a context, targeting tumor-specific upstream signaling could also theoretically offer higher safety, because of the capability of parallel and compensatory signaling to buffer unwanted cytotoxic effects in healthy cells^37^: in this perspective, MKK3 inhibitors have recently been developed^4,38^. Consistently, in line with tumor-specific increased MKK3 levels, we observed no toxic effects when targeting MKK3 in normal colonocytes, and we did not observe potentiation of 5-FU killing in these models, indicating MKK3 targeting, especially in combination with chemotherapy, could represent not only an effective but also a potentially safe therapeutic strategy to selectively kill cancer cells in CRC patients. In this perspective, our finding that high MKK3 levels are expressed especially in late stage CRC patients, which normally rely on chemotherapy as a therapeutic option^39^, prompts towards a rapid evaluation of MKK3-targeting potential in improving the therapeutic outcome in these patients.

## Materials and methods

### Cell culture

Authenticated CRC lines were obtained from ECACC: HT29 (ECACC 91072201), Colo205 (ECACC 87061208), Colo320DM (ECACC 87061205), SW480 (ECACC 87092801), and SW620 (ECACC 87051203). Authenticated primary human colonocytes were obtained from ATCC: CCD-18Co (ATCC CRL-1459) and CCD-841 CoN (ATCC CRL-1790). All lines were regularly monitored for mycoplasma contamination every 2 months by PCR^40^, and only mycoplasma-free cells were used for studies. All cells were used for no more than 15 passages. All cells were grown at 37 °C in HEPA-filtered humidified air in 5% CO_2_, in media supplemented with 10% heat-inactivated FBS (Gibco, Paisley, Scotland, UK, Cat. no. 10099-141), L-Glu (2 mM, Lonza, Burton on Trent, UK, Cat. no. 17-606E) and penicillin/streptomycin (100 U/ml, Gibco, Cat. no. DE17-602E). Colo205 and Colo320DM cells were grown in RPMI medium (Gibco, Paisley, Scotland, UK, Cat. no. 31870), HT29 were grown in McCoy’s medium (Lonza, Burton on Trent, UK, Cat. no. 12-688F), SW480 and SW620 were grown in L15 medium (Lonza, Burton on Trent, UK, Cat. no. BE12-700F), and HCT-116 (17) were grown in DMEM (Gibco, Paisley, Scotland, UK, Cat. no. 21885-025). CCD-18CO and CCD-841 primary cultures were grown in EMEM (Sigma-Aldrich, Saint Louis, MO, USA, Cat. no. M2279).

### Antibodies

Anti-phosphoMKK3/6 (1:1000, D8E9, Cell Signaling Technology, Danvers, MA, USA), anti-MKK3 (1:1000, C-19, Santa Cruz Biotechnology, Dallas, TX, USA), anti-MKK6 (1:1000, D31D1, Cell Signaling Technology), anti-phospho-p38 MAPK (1:1000, E1, Santa Cruz Biotechnology), anti-p38alpha MAPK (1:1000, 9F12, Santa Cruz Biotechnology), anti-p38beta MAPK (1:1000, F-3 Santa Cruz Biotechnology), anti-p38gamma MAPK (1:1000, E-4, Santa Cruz Biotechnology), anti-p38delta MAPK (1:1000, E-3, Santa Cruz Biotechnology), anti-p62/SQSTM1 (1:500, D-3, Santa Cruz Biotechnology), anti-LC3 (1:1000, Sigma Aldrich), anti-ERCC1 (1:1000, 8F1, Santa Cruz Biotechnology), anti-PARP (1:1000, Cell Signaling Technology) and anti-actin (1:1000, 13E5, Cell Signaling Technology) antibodies.

#### 5-FU IC50 determination

2 × 10^4^ cells were seeded in 24-well plates, and 24 h later, exposed for 6–24 h to 5-FU at a range of 5–40 μM concentrations. Cells were then washed in PBS and allowed to grow for 72 h. Number of viable cells was determined by crystal violet staining or Trypan blue exclusion assay.

#### MKK3 depletion and overexpression assays

MKK3 silencing was established in each line by engineering with doxycycline-inducible lentiviral-based system, as previously described^17^. All engineered sh/MKK3 and sh/SCR sublines were used for no more than 10 passages. The sh/RNA expression was obtained by exposing sh/SCR and sh/MKK3 sublines to 1 μg/ml doxycycline (Sigma Aldrich, Cat. no. D9891) for 72 h. For MKK3 overexpression, Colo205 cells were transduced with either empty (pCDNA3) or MKK3 encoding (pDNA3 HA-MKK3)^41^ vectors by Lipofectamine LTX and Plus reagent (Invitrogen, Carlsbad, CA, USA, Cat. no. 15338), according to manufacturer’s specifications, and experiments performed within three passages from transfection. For loss-of-function studies of p38delta MAPK and ERCC1 3 × 10^5^ cells, plated 24 h earlier, were transduced with 50 nM stealth RNAi oligos (Invitrogen, VHS40525, 10620318, and 10620319) or 30 nM siRNA (Ambion AM16708 ID 146876 and AM4611) with INTERFERin reagent (Polyplus, Illkirch-Graffenstaden, France, Cat. no. 409-10) according to manufacturer’s specifications, and lysates were collected 3 days after transduction.

#### Cell proliferation assays

sh/scr and sh/MKK3 sublines (1.5 × 10^6^ cells/100 mm dish) were exposed to doxycycline (1 μg/ml) for 48 h. Then seeded in 24-well plates (2 × 10^4^ cells/well) and further incubated for 120 h in presence of doxycycline before analyses. For 5-FU treatments, after plating in 24-well plate, as above, cells were incubated 24 h and then challenged with 5-FU IC50. After additional 24 h, cells were washed in PBS and allowed to grow for 72 h. Doxycycline was replaced every 48 h. Number of viable cells was determined by crystal violet staining or Trypan blue exclusion test.

### Western Blot analysis

sh/scr and sh/MKK3 sublines (1.5 × 10^6^ cells/100 mm dish) were pre-treated 72 h with doxycycline (1 μg/ml), then seeded in 60 mm dish (6.0 × 10^5^/dish) and further incubated 72 h with doxycycline before collection. For 5-FU treatment, cells were doxycycline pretreated 48 h as above described, then seeded in 60 mm dish (6.0 × 10^5^/dish) and 24 h later treated with *5-FU* IC50, then incubated further 24 h before collection. The collected cells were lysed in RIPA buffer (150 mM NaCl, 1% Triton X, 0.25% sodium deoxycholate, 0.1% SDS, 50 mM Tris/HCl, 20 mM EDTA, Complete mini protease inhibitor cocktail (Roche, Basel, Switzerland, Cat. no. 11836153001) 1 mM PMSF, 19 μg/ml Aprotinin, 50 mM NaF, 50 mM DTT, and 1 mM sodium orthovanadate), separated by SDS page and transferred to PVDF membranes (Immobilion-P, Merck-Millipore, Burlington, MA, USA, Cat. no. IPVH00010). Membranes were blocked in 5% BSA (AppliChem, Darmstadt, Germany, Cat. no. A1391) or nonfat-dried milk (Sigma Aldrich, Cat. no. M7409) and incubated overnight at +4 °C with specific antibodies. Actin was used as a loading control. Appropriate HRP-conjugated secondary antibodies (Biorad, Hercules, CA, USA, Cat. nos. 1721019 and 1706516) were used and immunocomplexes were visualized using ECL (GE Healthcare, Chicago, IL, USA, Cat. no. RPN2106). Densitometry was performed using ImageJ software.

#### p38 MAPK isoforms immunoprecipitation

Cells were seeded (1.5 × 10^6^ cells/100 mm dish) and treated 72 h with doxycycline, then challenged with *5-FU* IC50 for further 24 h and thereafter collected. Cells were lysed in RIPA buffer and immunoprecipitation performed by pre-clearing 500 μg of lysates with agarose protein G (Pierce, Waltham, MA, USA, Cat. no. 22852) and then precipitated using protein G-P38 isoform (above-described)/-isotype antibodies for 16 h. Beads were then washed in TBS, boiled in Laemmli buffer, and run on SDS/PAGE. Phospho-p38 and p38 isoform levels were then determined by Western Blot. Phosphop38/p38 isoform ratio was determined by densitometric analysis (Image J software) of phosphop38 and p38 isoform(s) expression levels.

#### Mice tumor xenografting and treatments

For in vivo experiments, either exponentially growing HT29 (1.0 × 10^6^ cell/mouse) or SW620 (2.0 × 10^6^ cell/mouse) cells were subcutaneously injected into the interscapular region of 45-day-old female nude mice (CD1 *nu/nu*, Charles River, Lecco, Italy). Eight animals/group were adopted based on previous in vitro and in vivo studies^17^. To deplete p38delta MAPK in xenograft lesions, stealth RNAi (1 mg/kg) was subcutaneously injected into the peritumoral region at specific time points (days 7, 9, 11, 21, 23, and 25) after inoculum. The 5-FU treatments (50 mg/kg)^17^ were administered by intraperitoneal injection on days 7, 9, 11, 21, 23, and 25. Tumor growth was assessed by caliper measurements twice a week and tumor volumes (TV) were estimated by the formula: TV = *a* × (*b*^2^)/2, where *a* and *b* are tumor length and width, respectively. Mice were euthanized one-week post-last treatment. At the end of the experimental procedure, all animals were sacrificed and tumors excised and analyzed by western blot to verify efficient p38delta MAPK depletion in vivo, and formalin fixed and paraffin embedded for histologic and immunohistochemical analyses. For quantification of necrosis, H&E sections from three tumors of each group were scored by a trained operator for the determination of the percentage of tumor tissue occupied by necrotic areas. Where available, different tumor fragments were individually scored to account for intratumor heterogeneity, and averaged. Results are summarized in Supplementary Table 3. All experiments with mice were executed in compliance with institutional guidelines and regulations and after approval from the appropriate institutional review board (141/2017-PR released on 02/13/2017) following the EU Directive 2010/63/EU at the Animal Technology Station, University of Rome “Tor Vergata”.

### Case selection, TMA construction, and IHC analysis

CRC samples were obtained from 185 patients, including 135 colon and 50 rectal carcinomas surgically treated at IRCCS Regina Elena National Cancer Institute between 2000 and 2013, were retrospectively evaluated. Tumors were staged according to the Union International Contre Cancer/tumor-node-metastasis system criteria^42^. All CRCs were histopathologically re-evaluated on haematoxylin and eosin-stained slides and representative areas were marked prior to tissue microarray (TMA) construction. Two core cylinders (1 mm diameter) were taken from selected CRCs and deposited into two separate recipient paraffin blocks using a specific arraying device (Alphelys, Euroclone, Milan, Italy). In seven cases, where informative results on TMA were absent due to missing tissue, no tumor tissue, or unsuccessful staining, were not scored and excluded from the final analysis. Two-micron sections of the resulting microarray block were made and used for immunohistochemical (IHC) analysis after transferring them to SuperFrost Plus slides (Menzel-Gläser, Braunschweig, Germany). IHC staining on TMA was performed using rabbit polyclonal antibody (Ab) anti-MKK3 (1:200, clone C19, Santa Cruz Biotechnology) in an automated immunostainer (Bond-III, Leica, Wetzlar, Germany). A pH 6 buffer was used as antigen retrieval for the antibody according to the manufacturer’s protocol. The levels of MKK3 were evaluated in terms of intensity of staining (0 = negative, 1+ = weak, 2+ = moderate, 3+ = strong) and percentage of positive cells. Images were obtained at ×20 magnification by using a light microscope (DM2000 LED, Leica) equipped with a software able to capture images. Tumor staging was performed by trained pathologists. Samples were part of the IRCCS Regina Elena National Cancer Institute Biorepository (BBIRE). Clinical details about the CRC patient samples included in the TMA, are summarized in Supplementary Fig. 1. The study was reviewed and approved by the ethics committee of the Regina Elena National Cancer Institute.

#### TCGA data analysis

TCGA data were interrogated using OncoLnc tool^43^. The OncoLnc tool identified a mean expression of 1864.38 (Arbitrary Units) for MKK3 mRNA expression in colon adenocarcinoma (COAD) patients. Patients were stratified into high MKK3 expressing (MKK3 mRNA expression >3200; *n* = 24) and low MKK3 expressing (MKK3 mRNA expression <1000; *n* = 33), and survival analysis at 2000 days was carried out by GraphPad Prism software.

#### Statistical analysis

Where appropriate, significance was evaluated using Student’s t-test, Fisher’s exact test, or two-way ANOVA. Statistical analysis was performed using GraphPad/PRISm software; *p < 0.05, **p < 0.01, ***p < 0.001, ****p < 0.0001.

## Supplementary information

Supplementary Figure 1

Supplementary Figure 2

Supplementary Figure 3

Supplementary Figure 4

Supplementary Figure 5

Supplementary Figure 6

Supplementary Figure 7

Supplementary Figure 8

Suppl. Table 1

Suppl. Table 2

Suppl. Table 3

Supplementary Figures and Tables Legends

RAW DATA
