# Supplementary figures and images for: MKK3 sustains cell proliferation and survival through p38DELTA MAPK activation in colorectal cancer
Source: Cell Death Dis. 2019 Nov 6;10(11):842. doi: 10.1038/s41419-019-2083-2 (PMC6834673; doi:10.1038/s41419-019-2083-2)

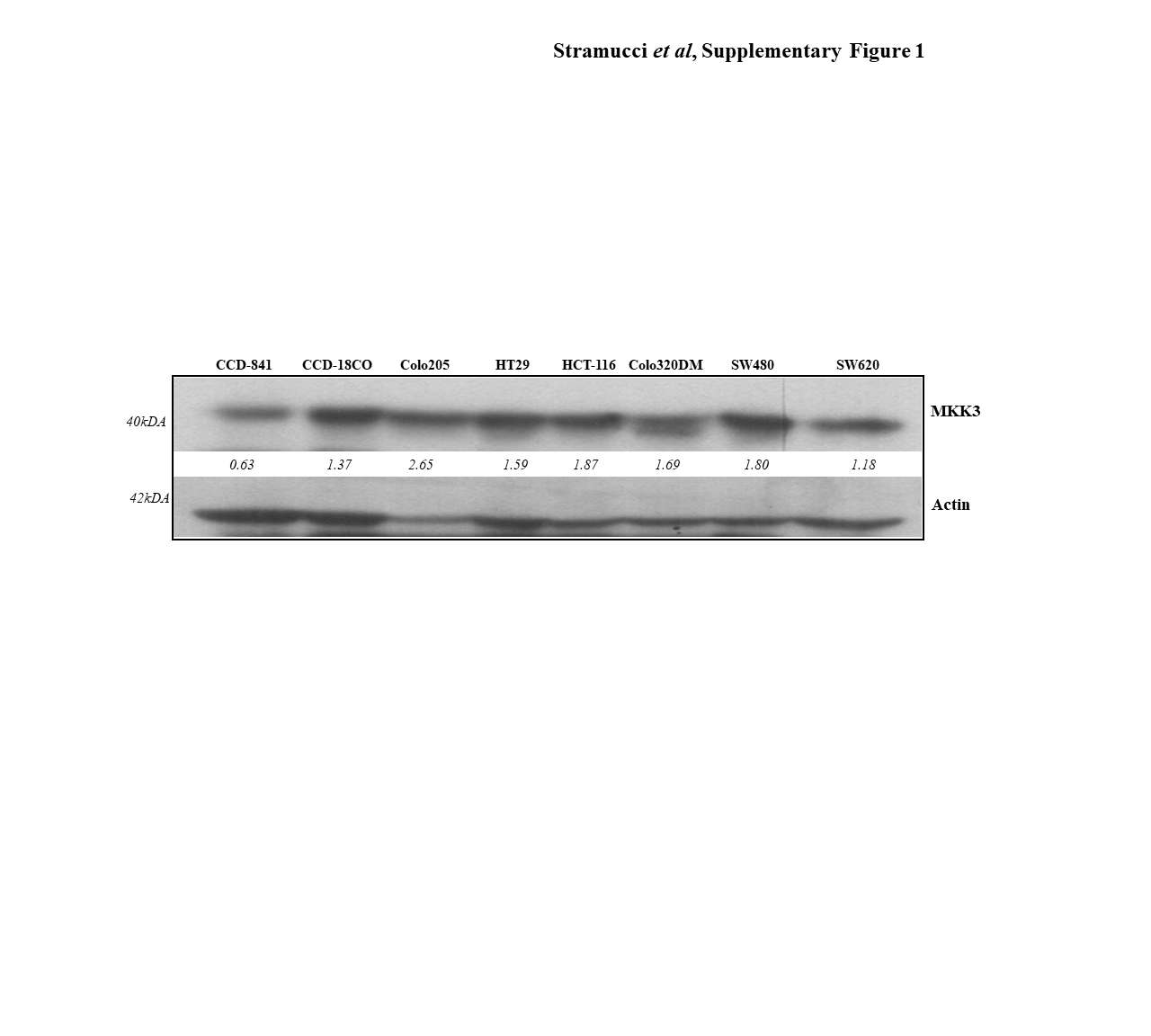

Supplement: Supplementary file 1 — Supplementary Figure 1 [file 41419_2019_2083_MOESM1_ESM.tif]

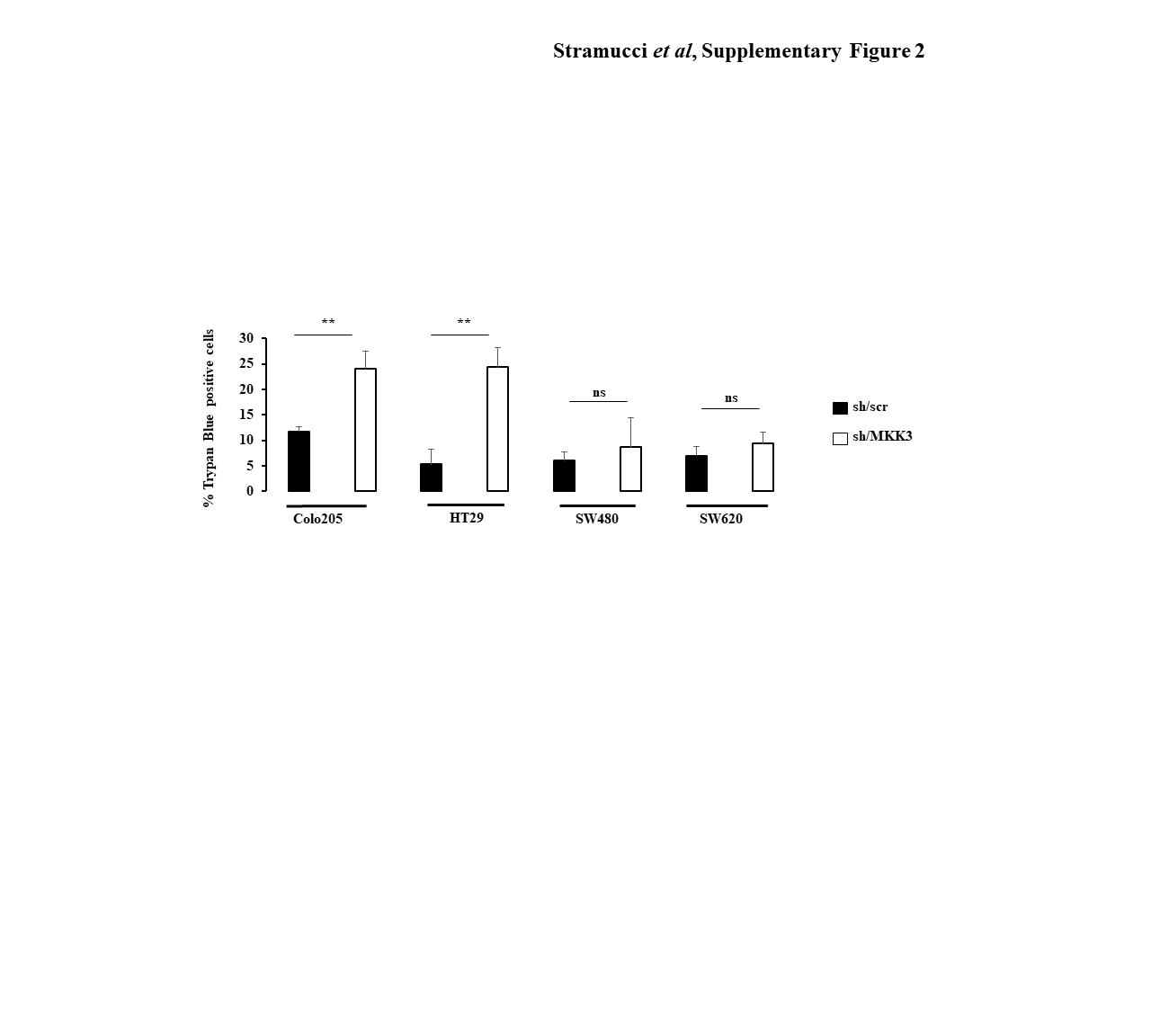

Supplement: Supplementary file 2 — Supplementary Figure 2 [file 41419_2019_2083_MOESM2_ESM.tif]

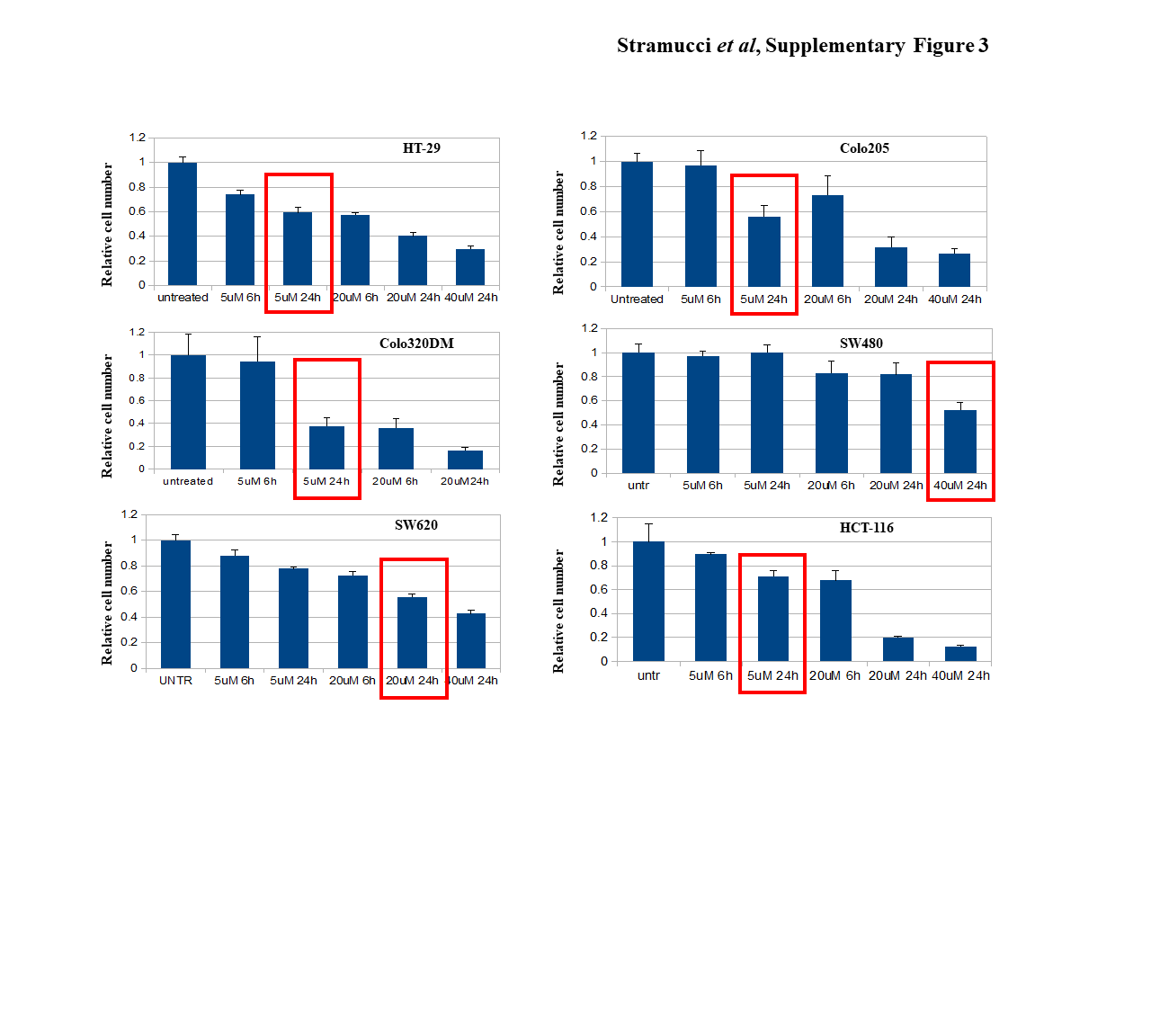

Supplement: Supplementary file 3 — Supplementary Figure 3 [file 41419_2019_2083_MOESM3_ESM.tif]

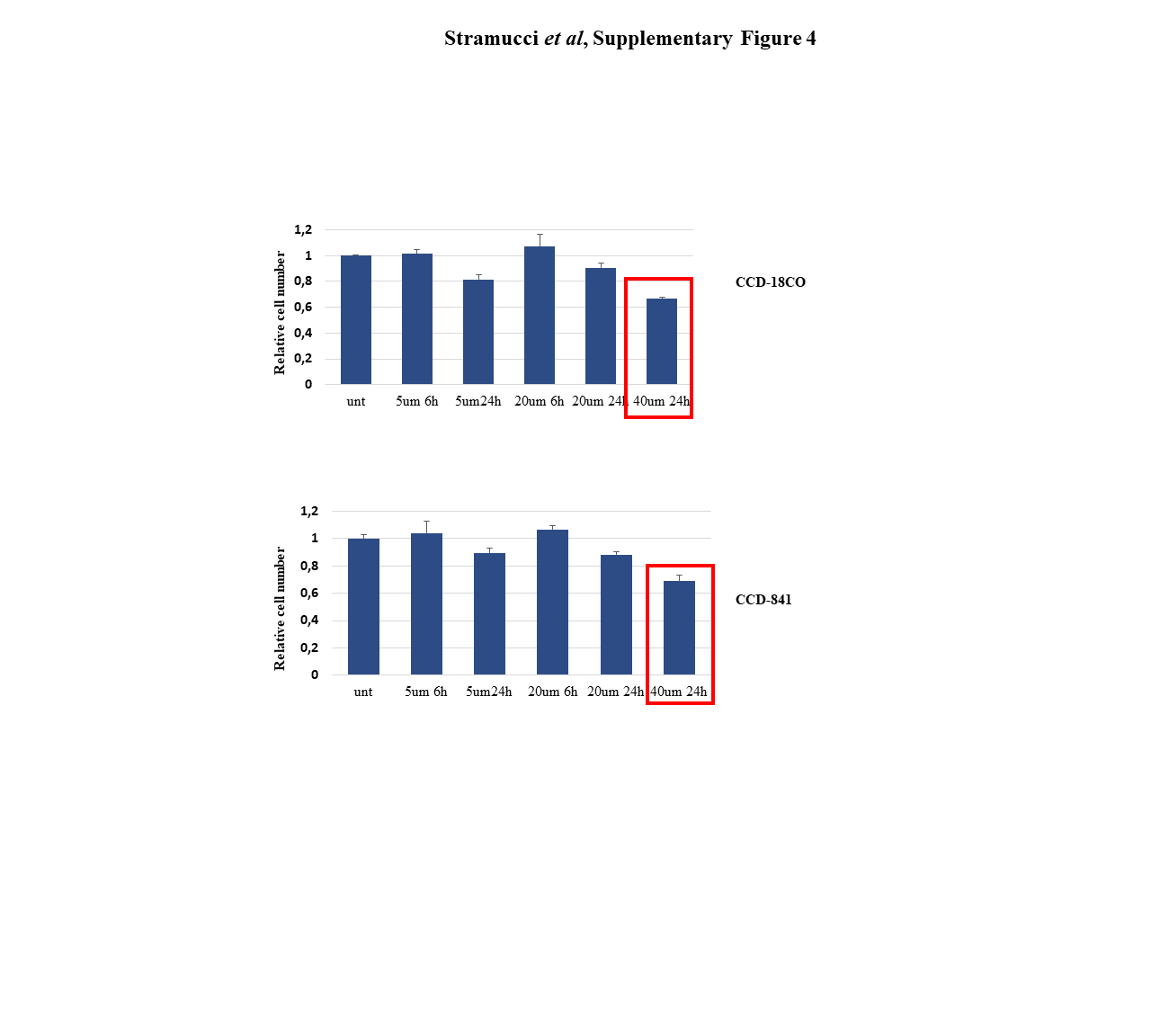

Supplement: Supplementary file 4 — Supplementary Figure 4 [file 41419_2019_2083_MOESM4_ESM.tif]

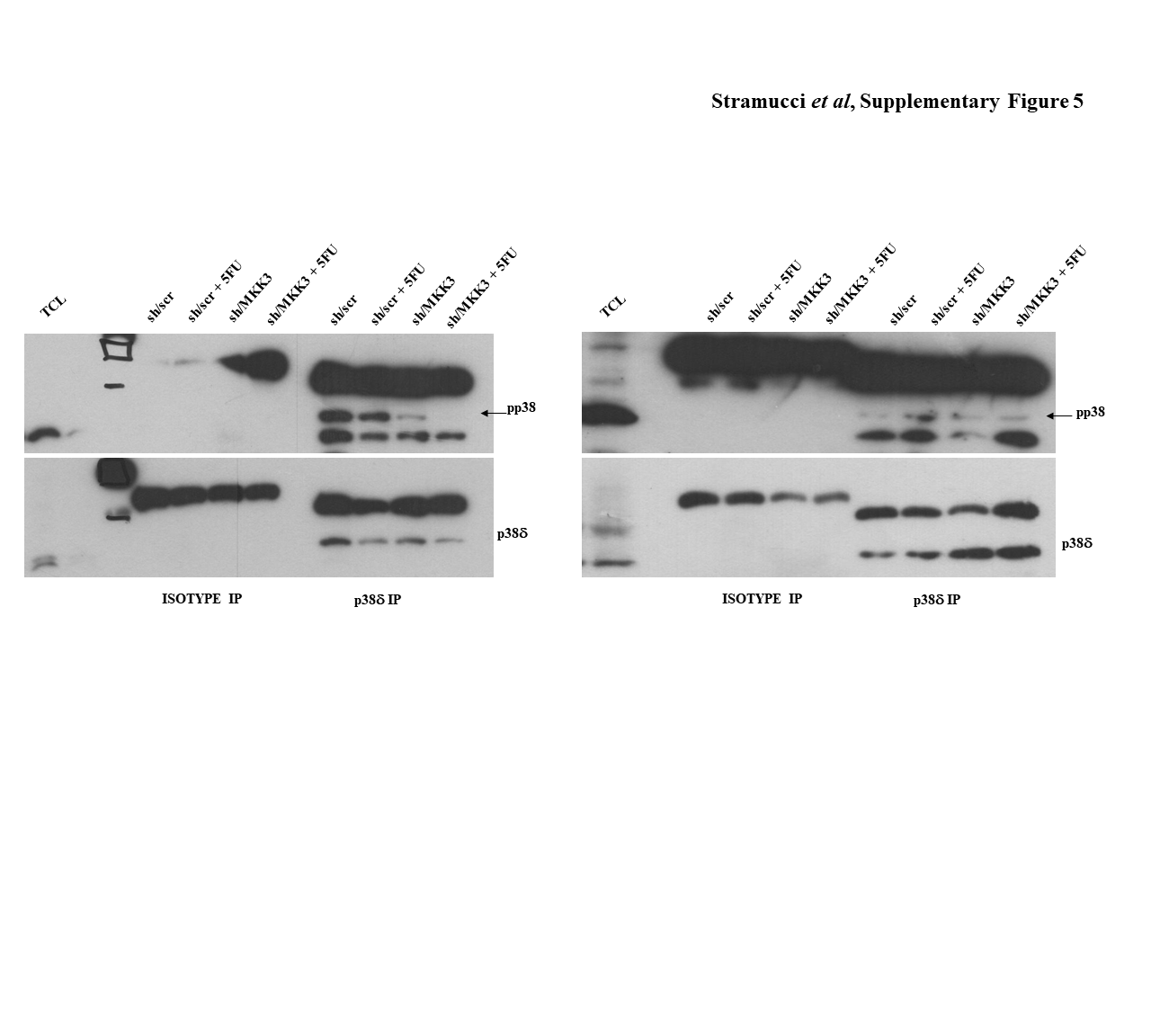

Supplement: Supplementary file 5 — Supplementary Figure 5 [file 41419_2019_2083_MOESM5_ESM.tif]

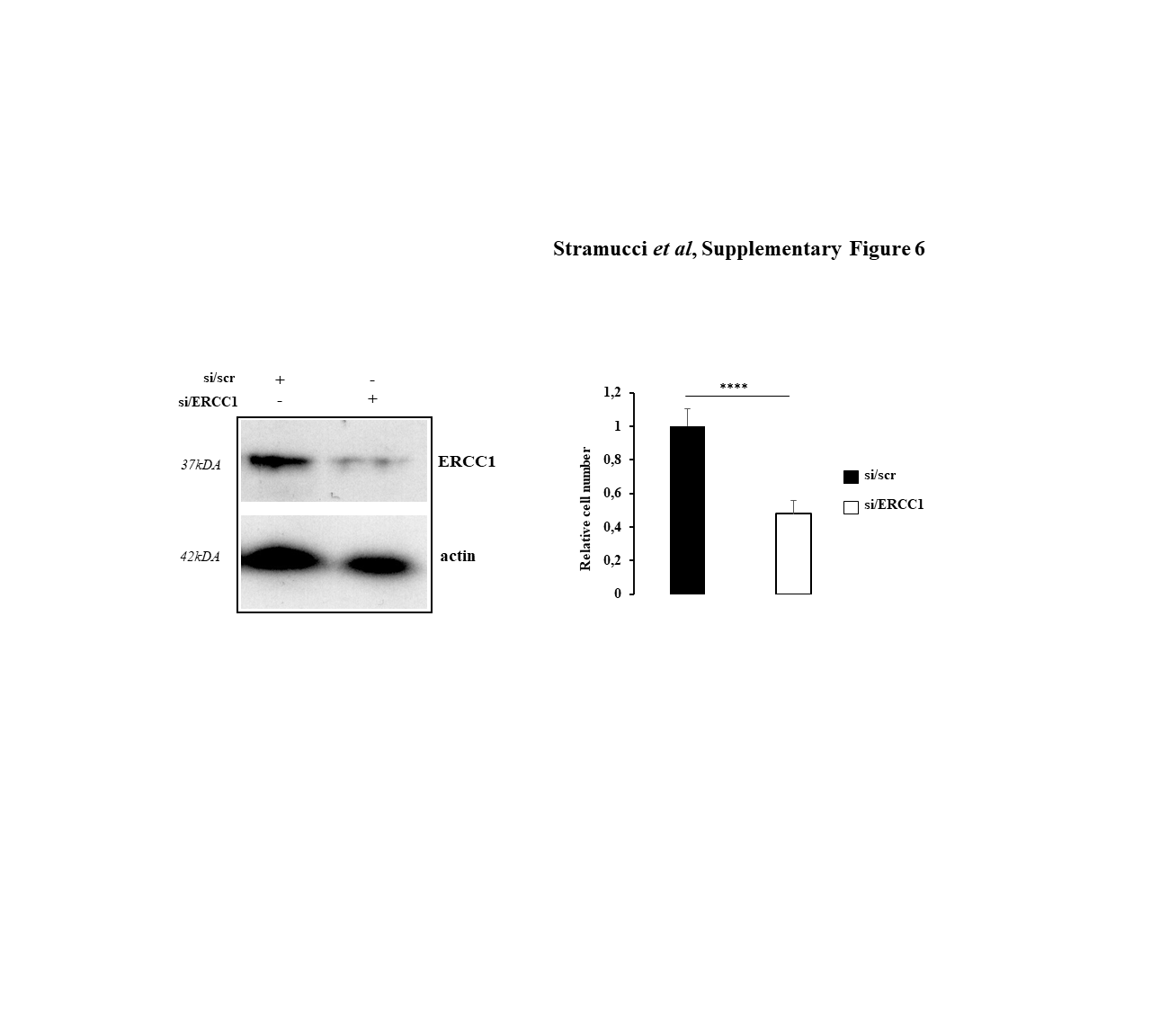

Supplement: Supplementary file 6 — Supplementary Figure 6 [file 41419_2019_2083_MOESM6_ESM.tif]

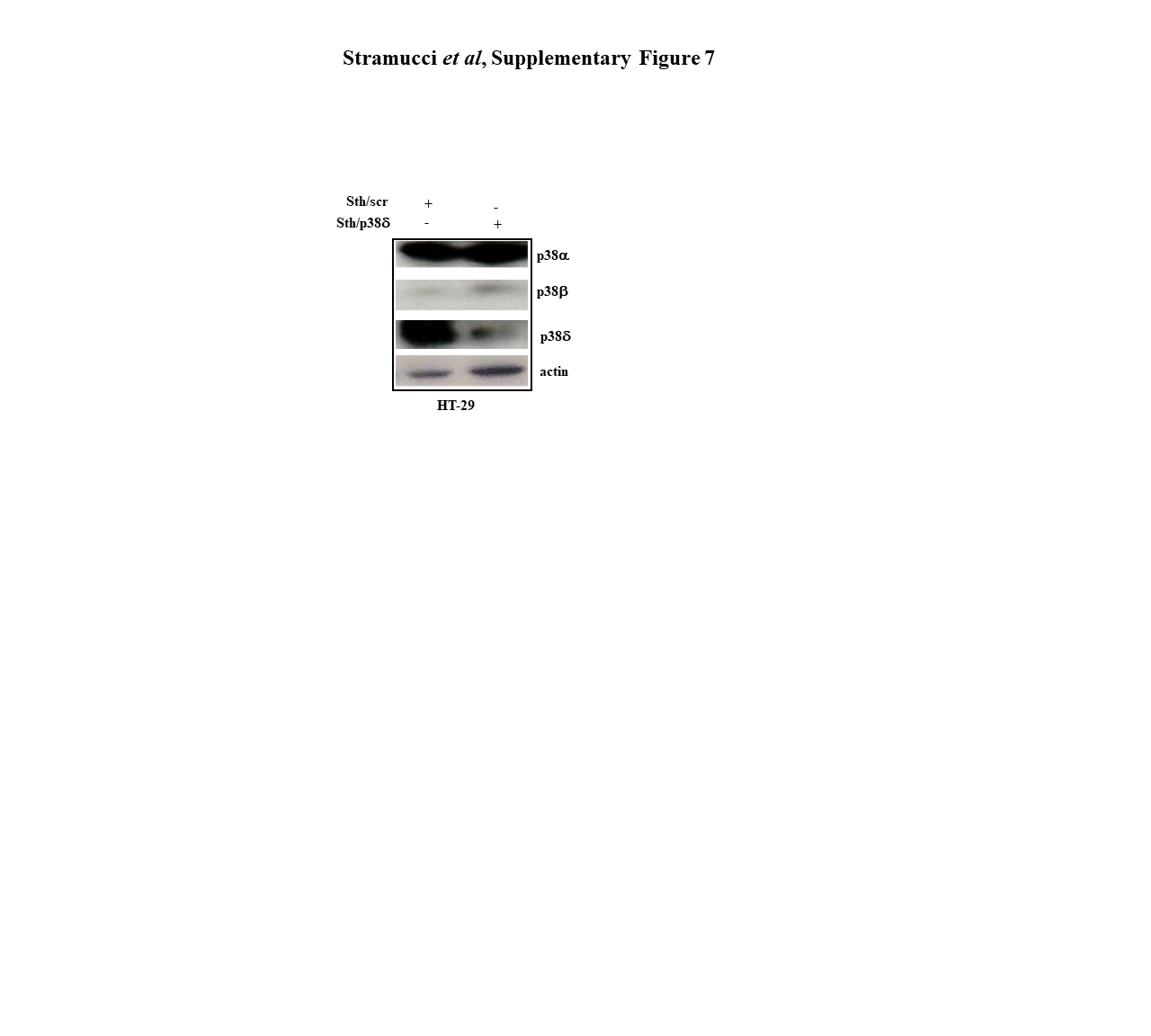

Supplement: Supplementary file 7 — Supplementary Figure 7 [file 41419_2019_2083_MOESM7_ESM.tif]

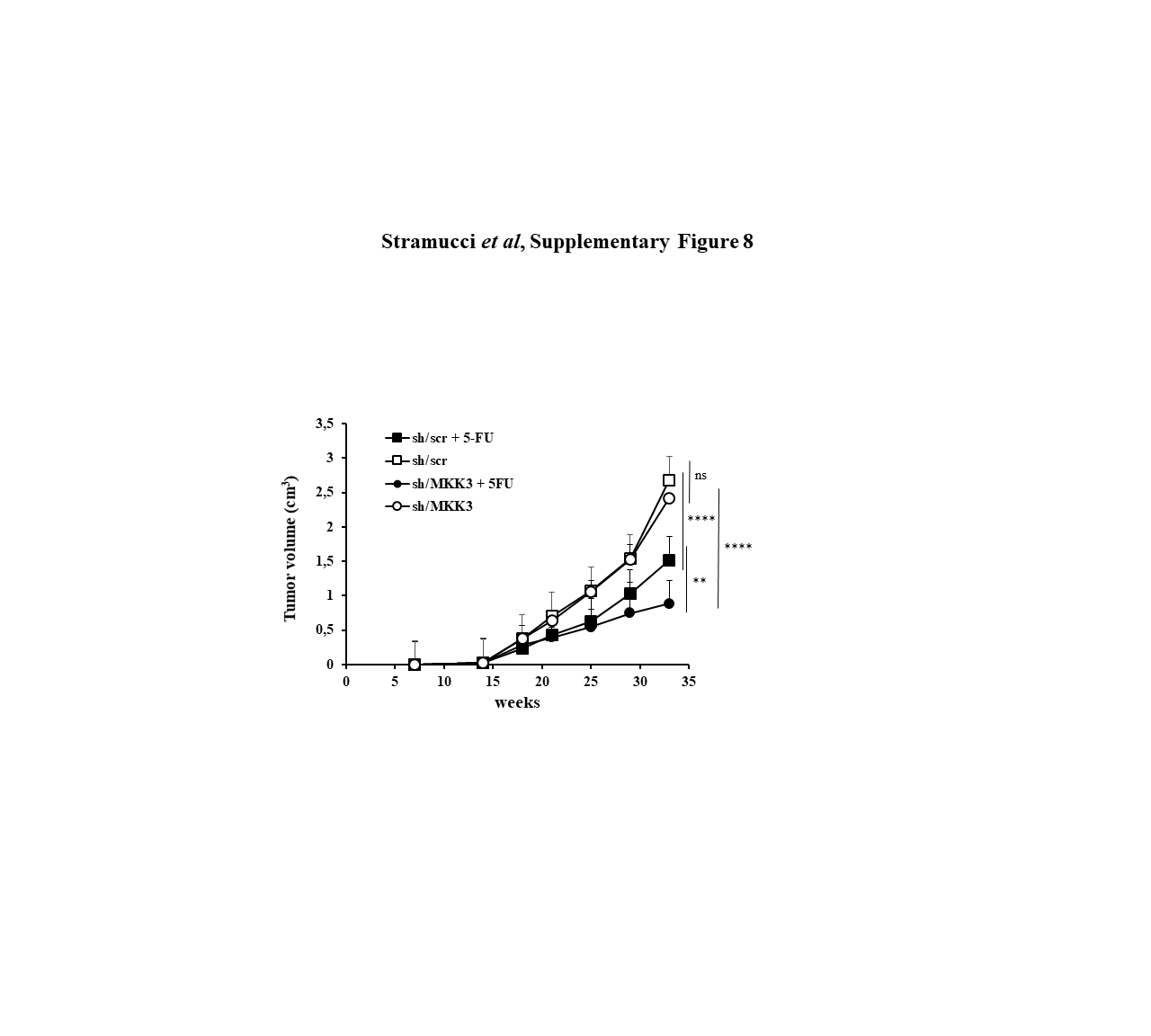

Supplement: Supplementary file 8 — Supplementary Figure 8 [file 41419_2019_2083_MOESM8_ESM.tif]

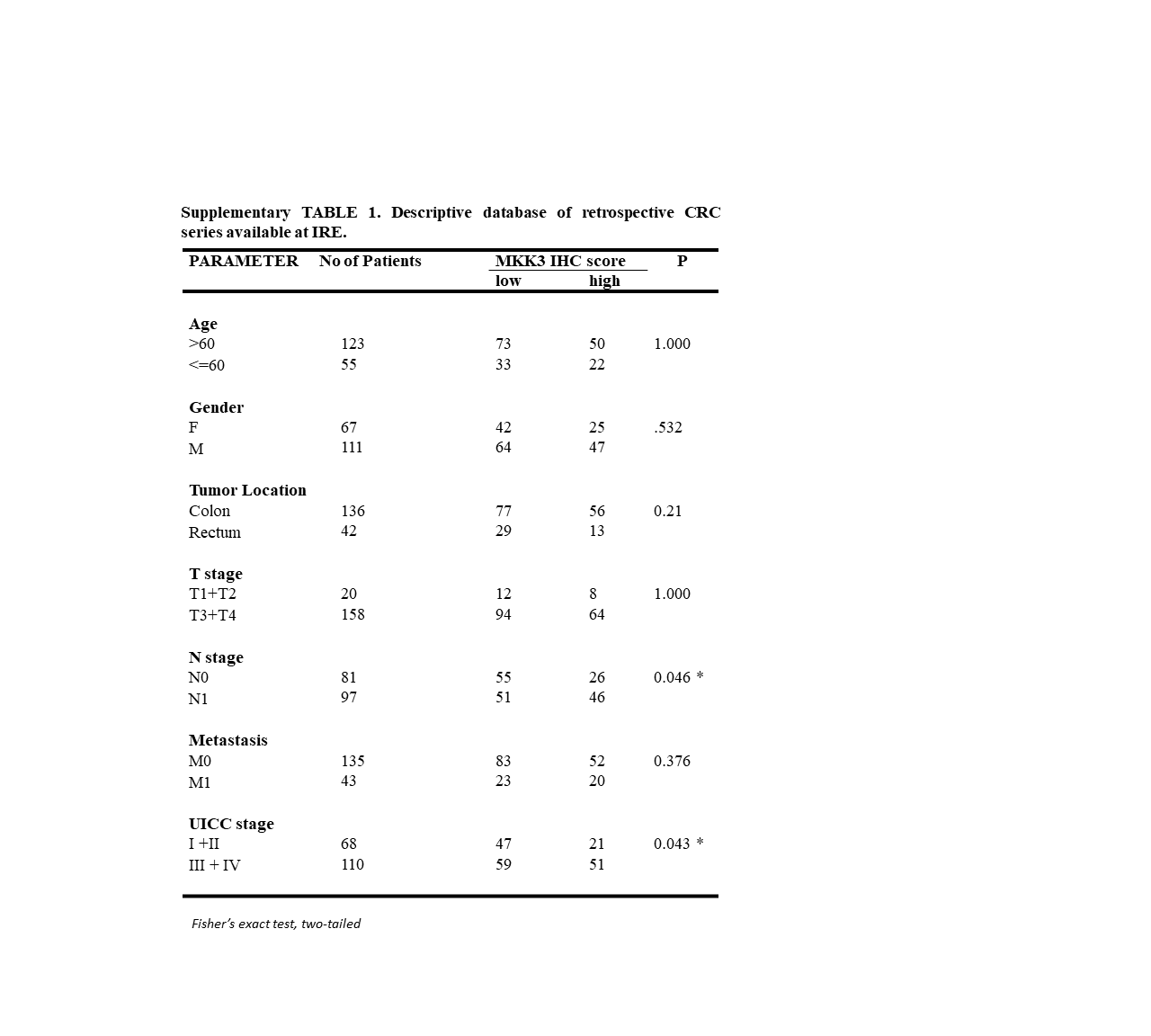

Supplement: Supplementary file 9 — Suppl. Table 1 [file 41419_2019_2083_MOESM9_ESM.tif]

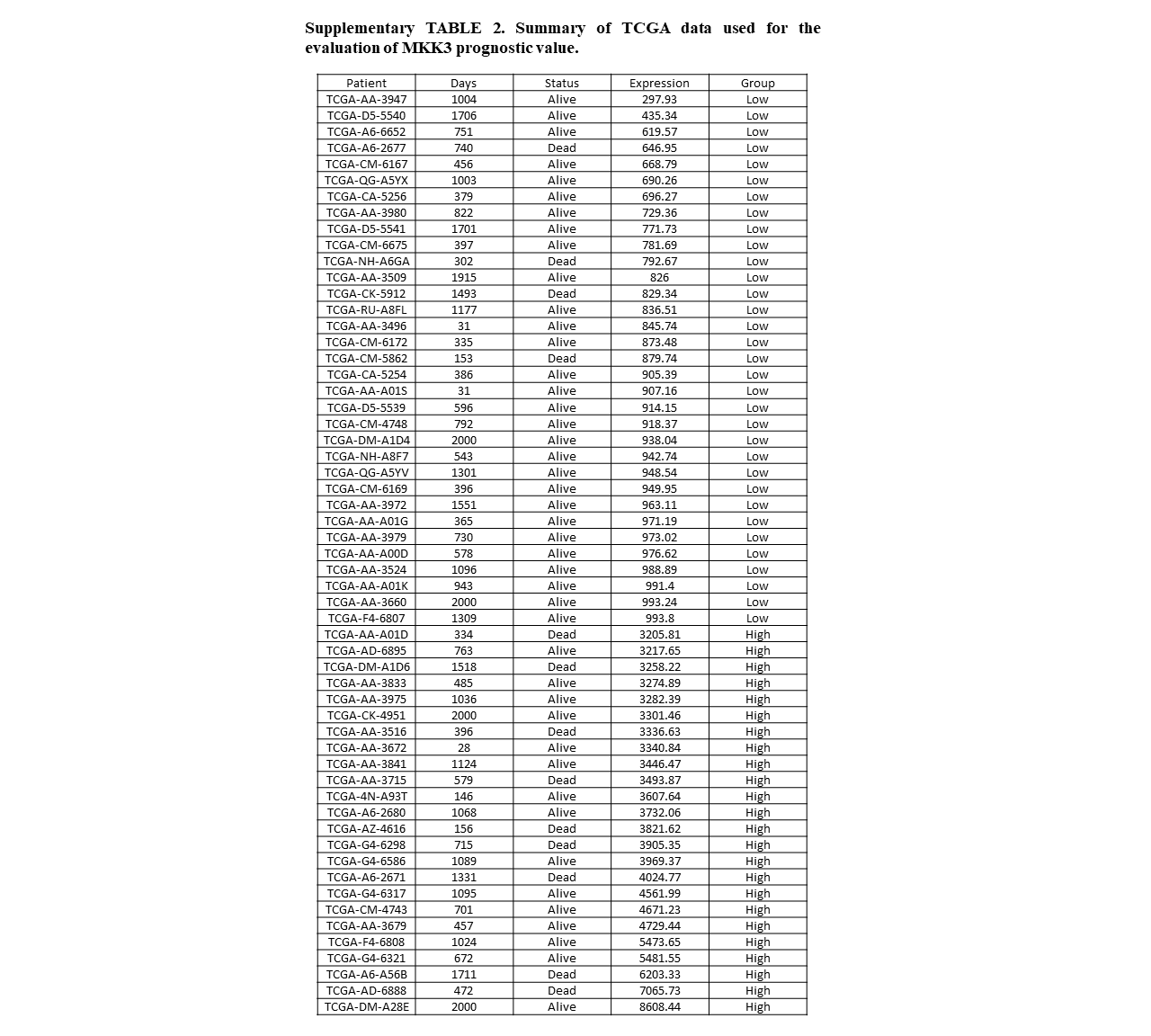

Supplement: Supplementary file 10 — Suppl. Table 2 [file 41419_2019_2083_MOESM10_ESM.tif]

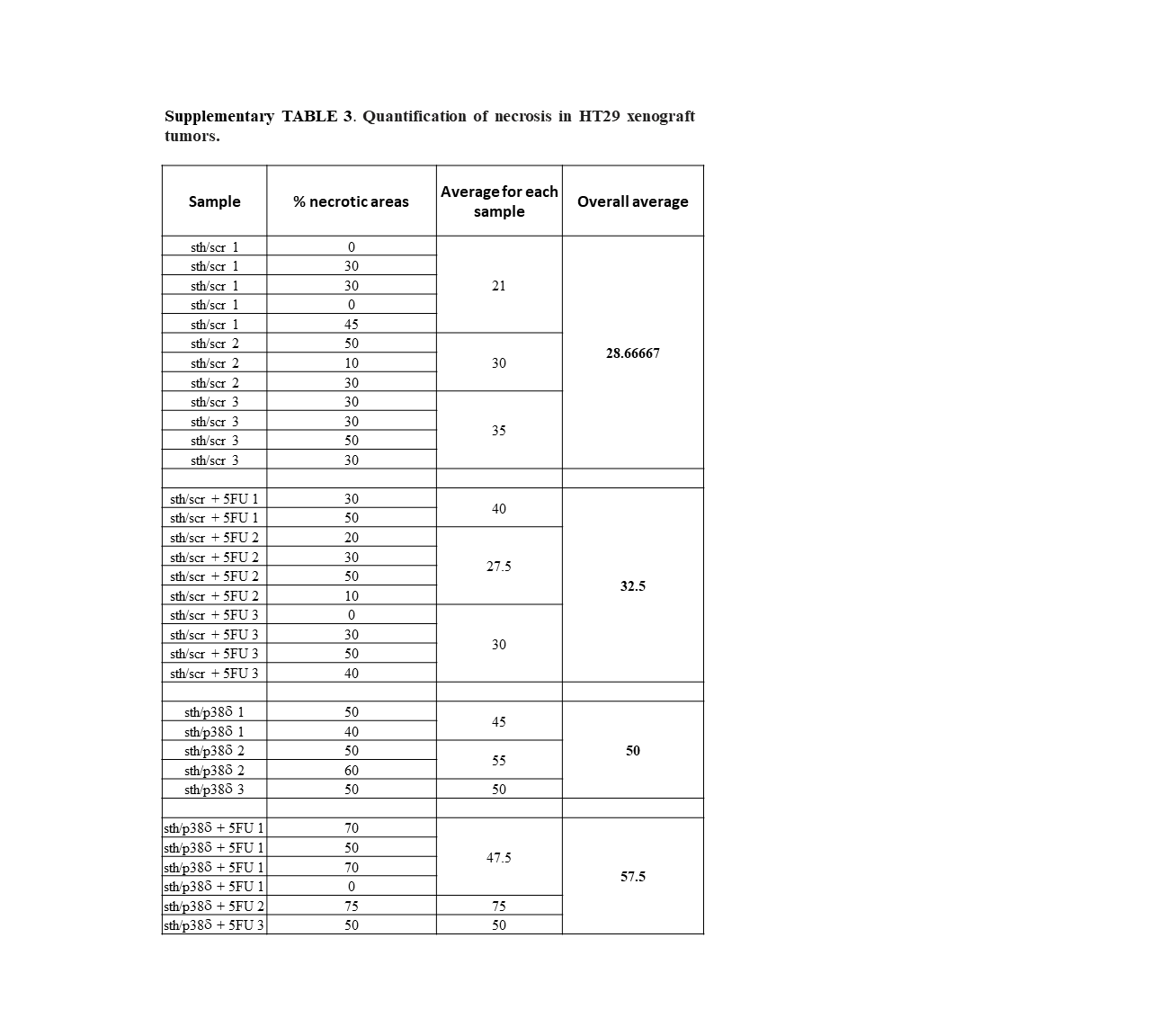

Supplement: Supplementary file 11 — Suppl. Table 3 [file 41419_2019_2083_MOESM11_ESM.tif]
