# Supplementary material for: MKK3 sustains cell proliferation and survival through p38DELTA MAPK activation in colorectal cancer
Source: Cell Death Dis. 2019 Nov 6;10(11):842. doi: 10.1038/s41419-019-2083-2 (PMC6834673; doi:10.1038/s41419-019-2083-2)
Supplement: Supplementary file 12 — Supplementary Figures and Tables Legends [file 41419_2019_2083_MOESM12_ESM.docx]

**SUPPLEMENTARY FIGURE LEGENDS.**

**Supplementary Figure 1.** **Evaluation of MKK3 levels in CRC and healthy colonocytes cells**. Representative Western Blot of MKK3 levels in CRC lines and primary colocytes cells. MKK3/actin ratio, as normalized to the average of the two healthy colonocytes cell lines, is shown.

**Supplementary Figure 2.** **MKK3 depletion affects tumor viability in CRC sensitive cells**. Viable cell number of sh/scr and sh/MKK3 sublines, after 144h doxycycline (1ug/ml) treatment, were determined by trypan blue exclusion assay and numbers of stained cells reported as mean and S.D. of three independent experiments. Significance was analyzed using unpaired t-test. **p<0.01, no significant.

**Supplementary Figure 3. 5-FU drug-dosage determination for CRC lines.** Relative cell number (as compared to untreated control) of CRC cells 72h after exposure to different 5-Fluorouracyl concentration and time. A dosage allowing for 30-70% reduction in cell-growth (red box) was selected for molecular and functional characterization.

**Supplementary Figure 4. 5-FU drug-dosage determination in primary colonocytes.** Relative cell number (as compared to untreated control) of normal colonocytes 72h after exposure to different 5-Fluorouracyl concentration and time. A dosage allowing for 30-70% reduction in cell-growth (red box) was selected for molecular and functional characterization as performaed for CRC lines.

**Supplementary Figure 5. MKK3 silencing prevents 5-FU induced p38delta phosphorylation.** Representative pp38 (upper) and p38delta (lower) western blots of Immunoprecipitates from sh/scr and sh/MKK3 HT-29 (left) and SW620 (right) cells exposed or not to 5-FU.

**Supplementary Figure 6. ERCC1 depletion affects tumor viability in CRC cells.** Left panel, western Blot on protein lysates from si/scr and si/ERCC1 transfected Colo205 cells. Blots were incubated with indicated antibodies, and more relevant bands from the same filter at same exposure length are reported. Representative results of three independent experiments are reported; right panel, relative cell number of si/scr and si/ERCC1 transfected Colo205 cells. Data are reported as mean and S.D. of results from three independent experiments and analyzed using unpaired t-test. ****<0.0001.

**Supplementary Figure 7. The sth/p38d selectively ablates p38d MAPK isoform.** The p38a, b and d MAPK protein levels in HT-29 cell transduced with either sth/scr or sth/p38delta oligonucleotides (50nM). Blots were incubated with indicated antibodies, and more relevant bands from the same filter at same exposure length are reported. Representative results of three independent experiments are reported.

**Supplementary Figure 8. MKK3 depletion boosts 5FU response in xenografted SW620 tumors.** Exponentially growing SW620 sh/scr or sh/MKK3 sublines (2.0×106 cell/mouse) were implanted s.c. in CD1 nu/nu female 45 days old mice. After tumor nodule formation, animals were randomized (8 mice / group), and DOX (2.0 g/l, tap water) delivered to all mice replacing every three days. 5FU treatments were performed according to schedule as in Fig.6A. Tumor growth was followed by caliper measurements twice a week. Representative data of two independent experiments are reported. Data were analyzed using two-way ANOVA. *p<0.05, **p<0.01, ***p<0.001, ****<0.0001, ns: no significant.

**Supplementary TABLE 1. Descriptive database of retrospective CRC series available at IRE.** Relative frequency for T, N, M, as well as grade (G) and tumor stage and recurrence of disease with a median follow-up of 66 months (95% CI 61.8–71.5), for CRC patient samples used in the TMA analysis.

**Supplementary TABLE 1. Descriptive database of retrospective CRC series available at IRE.** Relative frequency for T, N, M, as well as grade (G) and tumor stage and recurrence of disease with a median follow-up of 66 months (95% CI 61.8–71.5), for CRC patient samples used in the TMA analysis.

**Supplementary TABLE 3. Quantification of necrosis in HT29 xenograft tumors.** H&E sections from 3 tumors for each group were scored for the determination of the percentage of tumor tissue occupied by necrotic areas. Where available, different tumor sections were individually scored to account for intratumor heterogeneity and averaged.
