# Supplementary material for: MKK3 sustains cell proliferation and survival through p38DELTA MAPK activation in colorectal cancer
Source: Cell Death Dis. 2019 Nov 6;10(11):842. doi: 10.1038/s41419-019-2083-2 (PMC6834673; doi:10.1038/s41419-019-2083-2)

Figure 2b

Colo205

P62 MKK3 Actin

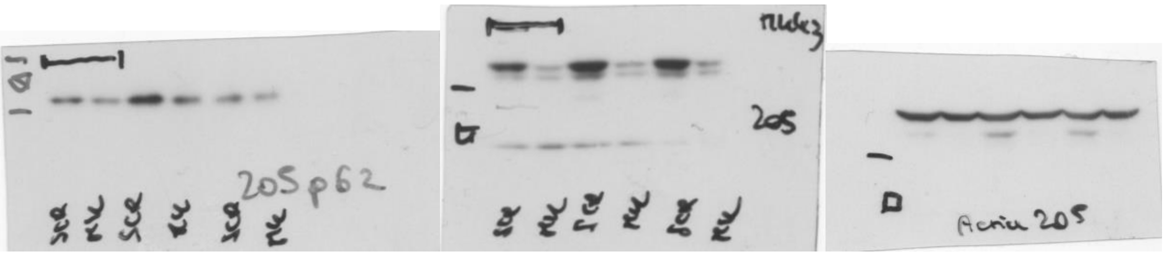

HCT-116

P62 MKK3 Actin

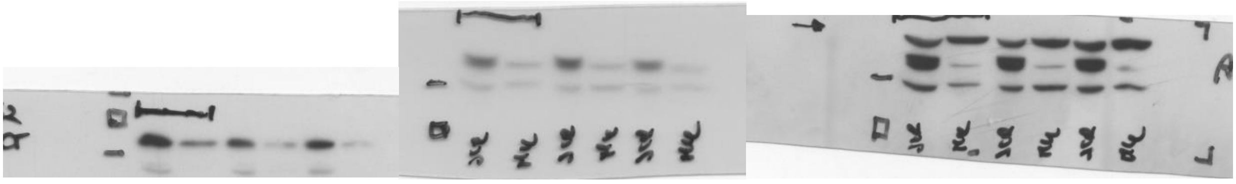

HT-29

P62 MKK3 Actin

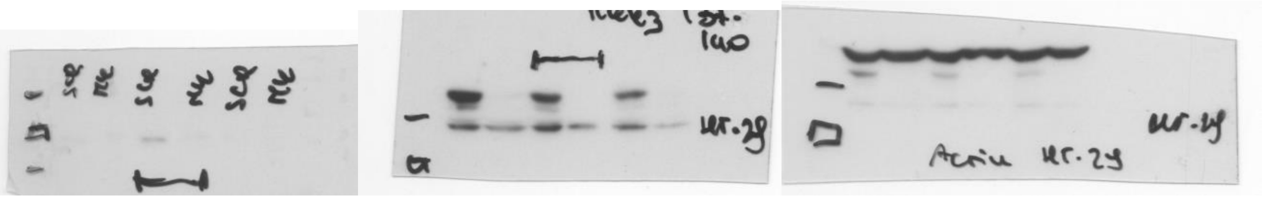

SW480

P62 MKK3 Actin

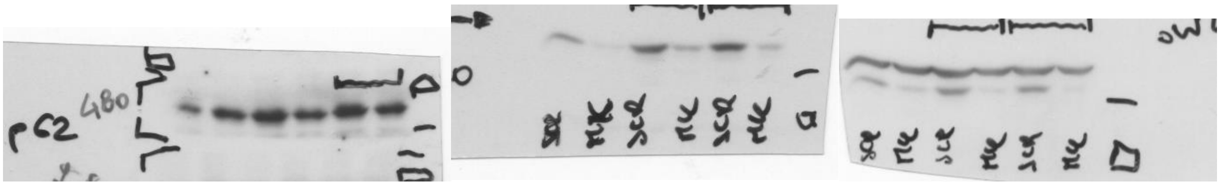

SW620

P62 MKK3 Actin

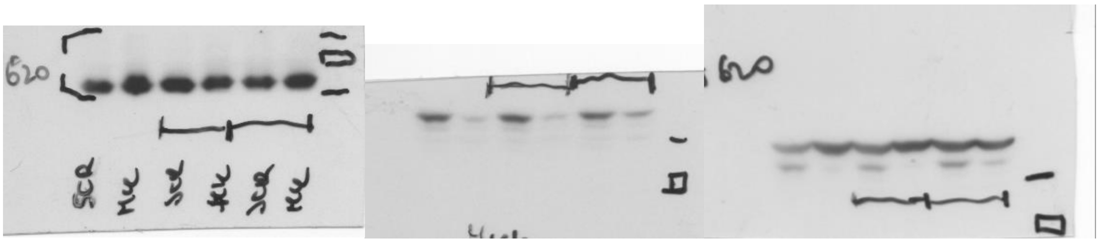

CCD841 and CCD18

P62 MKK3 and Actin

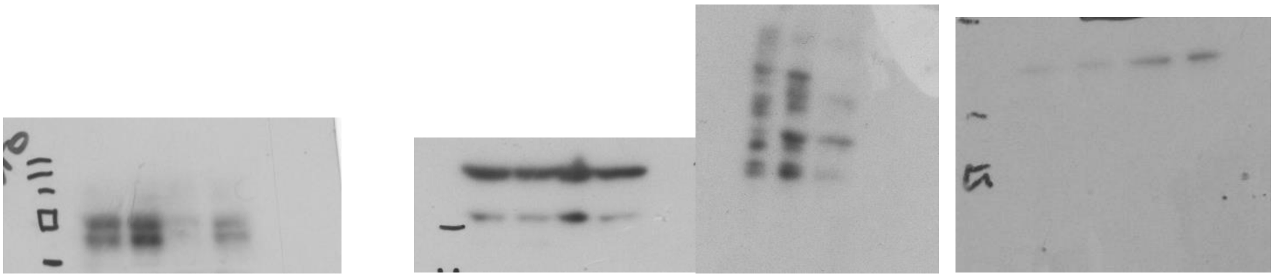

Figure 2C

LC3

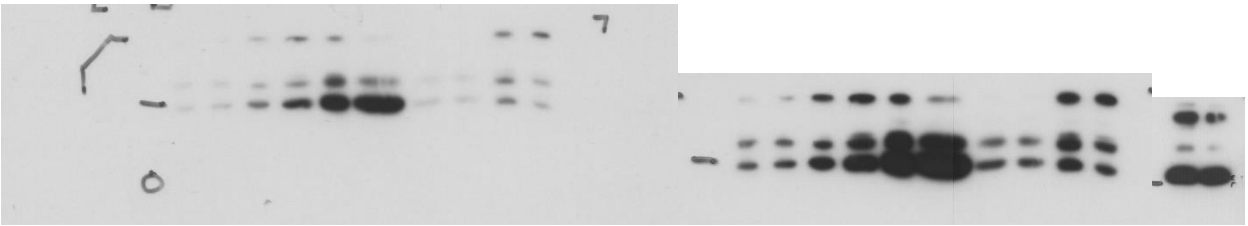

Actin

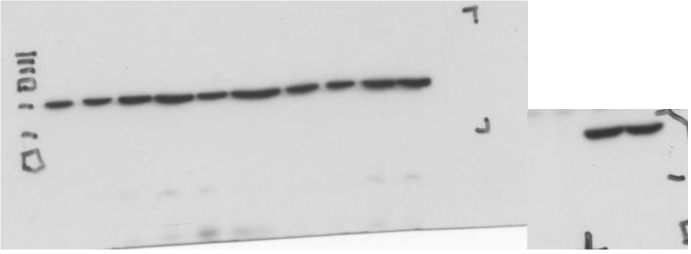

Figure 3b

Actin MKK3

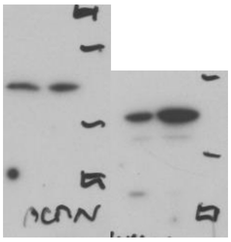

Figure3C

Colo205

pMKK3/6 MKK3 MKK6 Actin

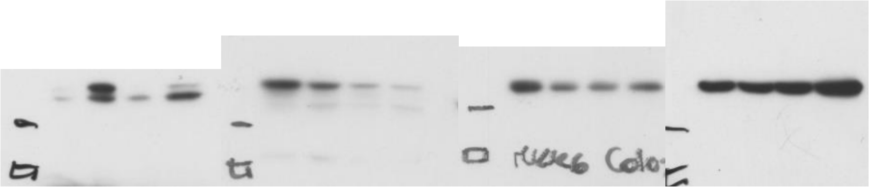

HCT116

pMKK3/6 MKK3 MKK6 Actin

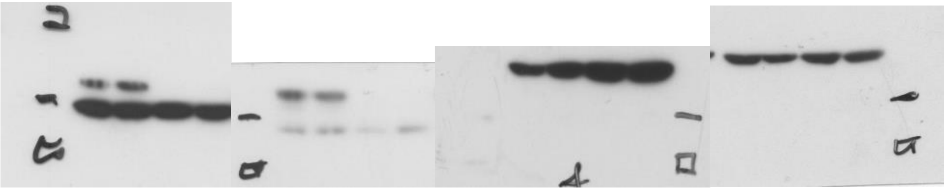

HT-29

pMKK3/6 MKK3 MKK6 Actin

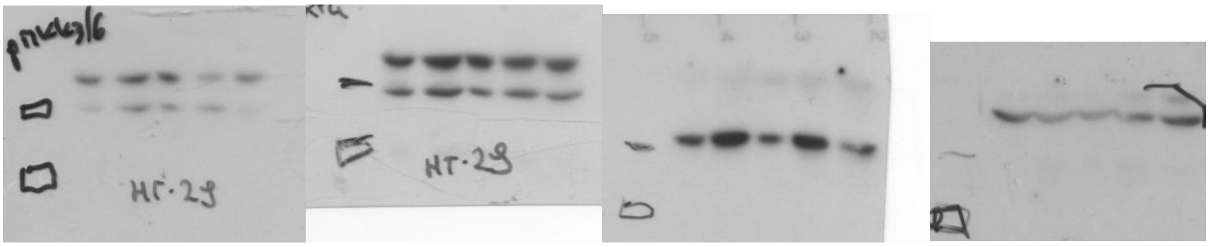

SW480

pMKK3/6 MKK3 MKK6 Actin

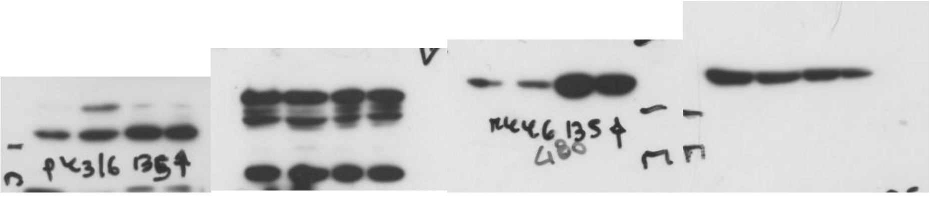

SW620

pMKK3/6 MKK3 MKK6 Actin

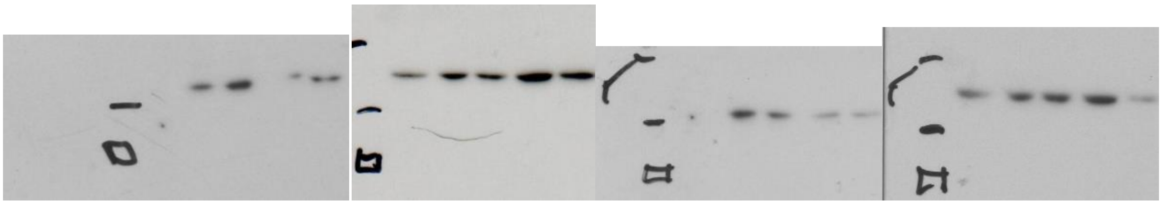

CCD18CO and CCD841

pMKK3/6 MKK3

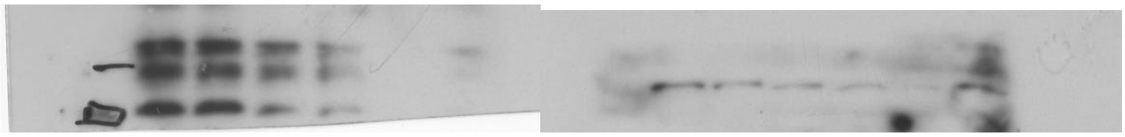

MKK6 Actin

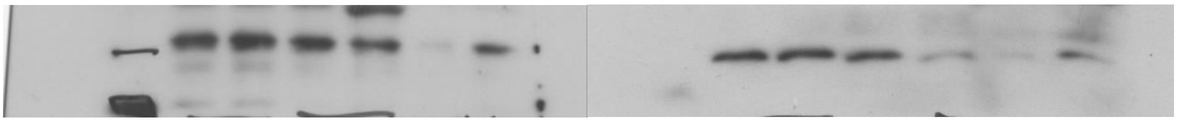

Figure 4A

phosphoP38

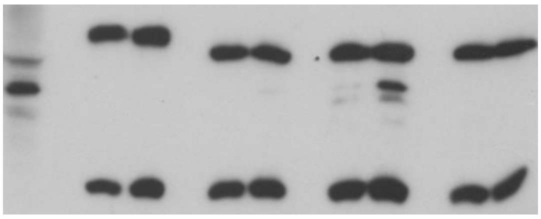

p38 alfa and delta

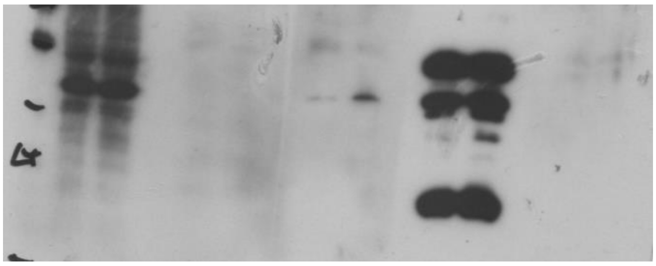

P38 total

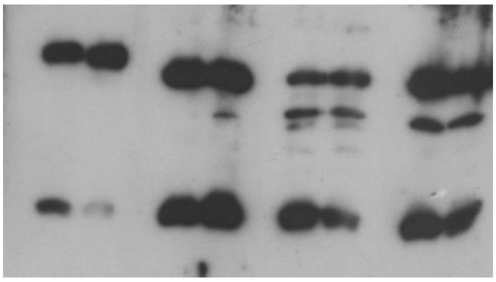

Figure 4B

phosphoP38

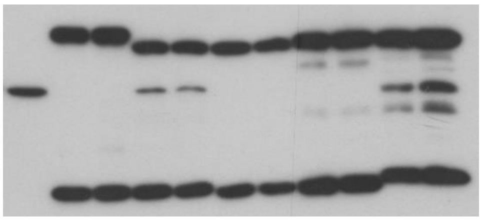

P38 total

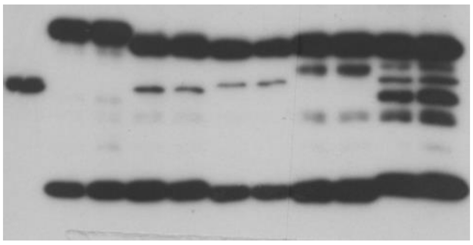

Figure 5A

HT29

pMKK3/6 MKK3 ERCC1 Actin

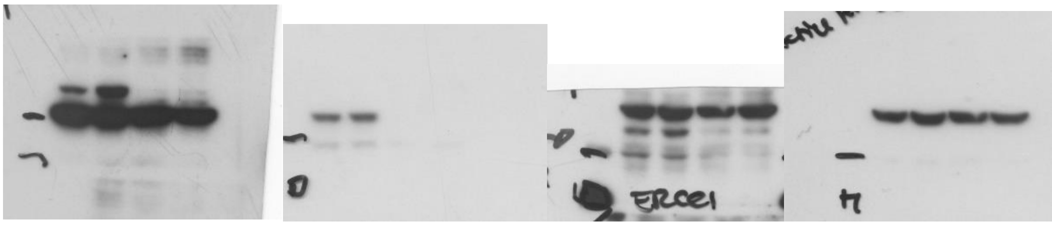

SW620

pMKK3/6 MKK3 ERCC1 Actin

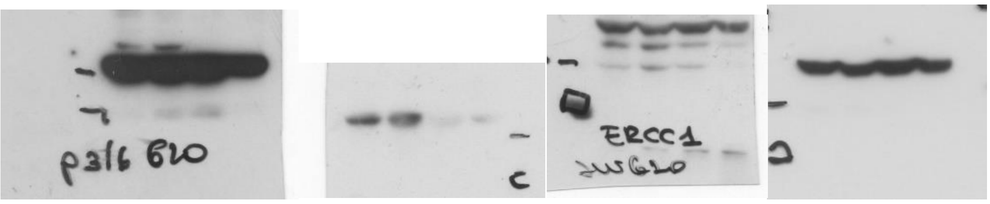

Figure 5B

HT29

P38delta p62 ERCC1 Actin

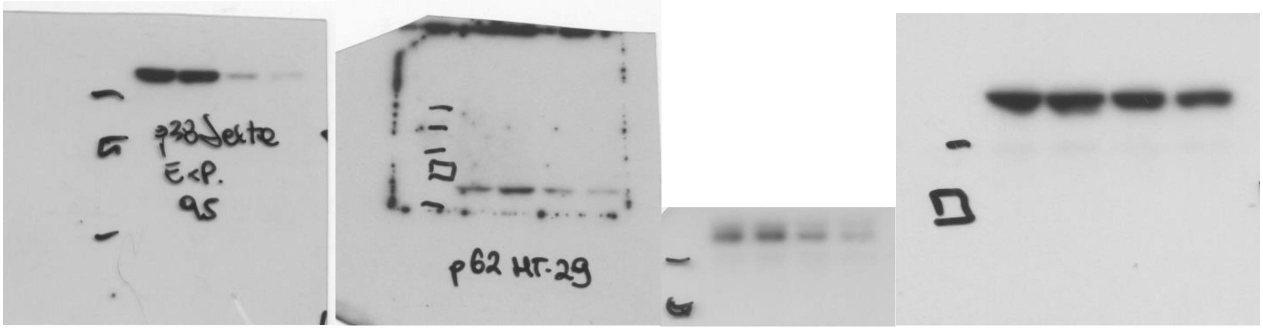

SW620

P38delta p62 ERCC1 Actin

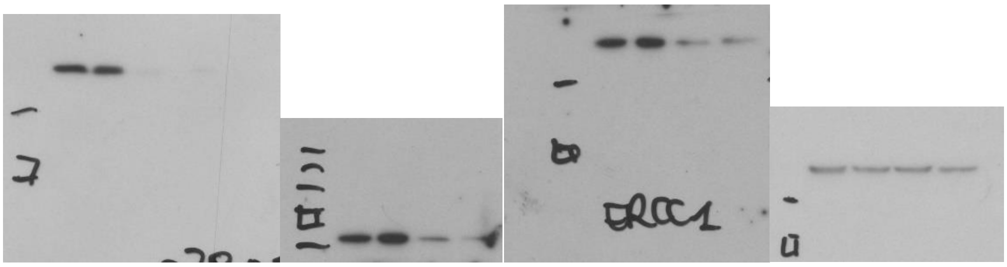

Figure 6C

PARP

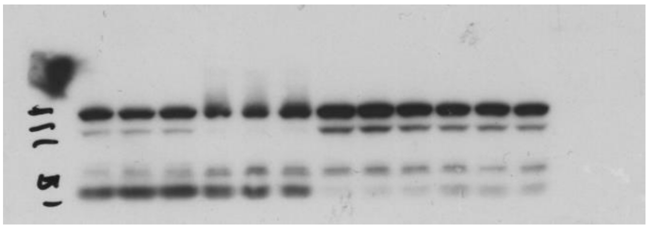

P38delta

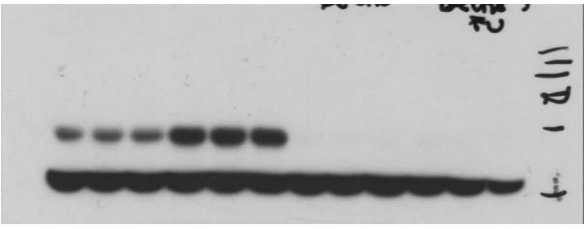

P62 and ERCC1

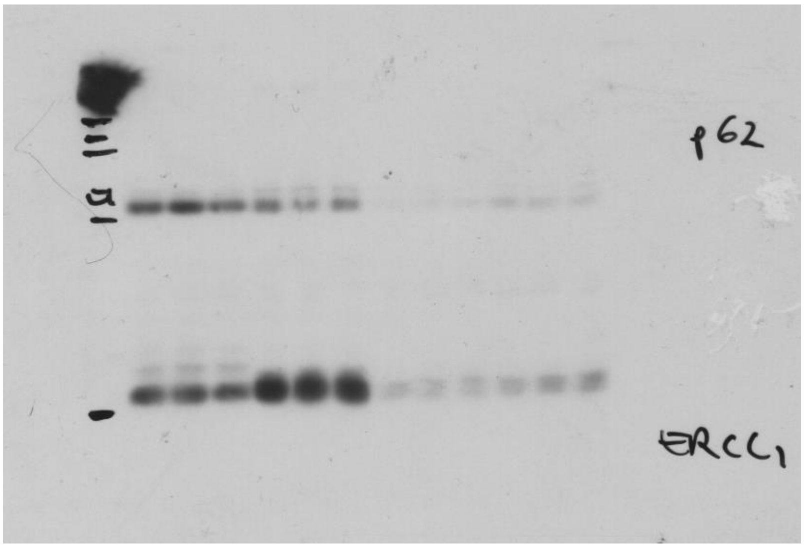

Actin

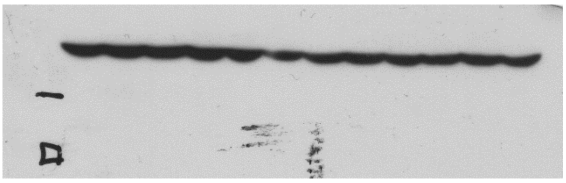

Supplement: Supplementary file 13 — RAW DATA [file 41419_2019_2083_MOESM13_ESM.pdf]
